# Identification and validation of a combined hypoxia and immune index for triple‐negative breast cancer

**DOI:** 10.1002/1878-0261.12747

**Published:** 2020-07-01

**Authors:** Shaoquan Zheng, Yutian Zou, Jie‐ying Liang, Weikai Xiao, Anli Yang, Tiebao Meng, Shilin Lu, Zhongbing Luo, Xiaoming Xie

**Affiliations:** ^1^ Department of Breast Oncology Sun Yat‐sen University Cancer Center Guangzhou China; ^2^ State Key Laboratory of Oncology in South China Collaborative Innovation Center for Cancer Medicine Sun Yat‐sen University Cancer Center Guangdong China; ^3^ Department of Medical Oncology Sun Yat‐sen University Cancer Center Guangzhou China; ^4^ Department of Breast Cancer Cancer Center Guangdong Provincial People's Hospital Guangdong Academy of Medical Sciences Guangzhou China; ^5^ Department of Radiology Sun Yat‐sen University Cancer Center Guangzhou China; ^6^ Zhongshan School of Medicine Sun Yat‐Sen University Guangzhou China; ^7^ Department of Breast Surgery First Affiliated Hospital of Gannan Medical College Ganzhou City China

**Keywords:** hypoxia, immune, immunotherapy, triple‐negative breast cancer

## Abstract

The interaction between hypoxia and immune status has been confirmed in various cancer settings, and corresponding treatments have been investigated. However, reliable biomarkers are needed for individual treatment, so we sought to develop a novel scoring system based on hypoxia and immune status. Prognostic hypoxia–immune status‐related signatures of patients with triple‐negative breast cancer (TNBC) were identified in The Cancer Genome Atlas (TCGA) (*N* = 158), Molecular Taxonomy of Breast Cancer International Consortium (METABRIC) (*N* = 297), and GSE58812 (*N* = 107). LASSO Cox regression was used for model construction. Hypoxia and immune status expression profiles were analyzed, and infiltrating immune cells were compared. Quantitative real‐time PCR (qRT‐PCR) was used for validation in the Sun Yat‐sen University Cancer Center (SYSUCC) cohort, and immunofluorescence was applied for the detection of hypoxia and immune markers in cancer tissues. Ten cross‐cohort prognostic hypoxia–immune signatures were included to construct the comprehensive index of hypoxia and immune (CIHI) in the METABRIC cohort. Two subgroups of patients with distinct hypoxia–immune status conditions were identified using CIHI: hypoxia^high^/immune^low^ and hypoxia^low^/immune^high^, with a significantly better overall survival (OS) rate in the latter (*P* < 0.01). The prognostic value of CIHI was further validated in the TCGA, GSE58812, and SYSUCC cohorts (*P* < 0.01). Hypoxia–immune signatures were significantly differentially expressed between the two groups, and more active immune responses were observed in the hypoxia^low^/immune^high^ group. Cytotoxic lymphocytes were inversely correlated with CIHI in silico. Differentially expressed CA‐IX and stromal PD‐L1 were detected between subgroups of the SYSUCC cohort. A hypoxia–immune‐based cross‐cohort classifier for predicting prognosis was developed and validated, which may guide hypoxia modifier treatment and immunotherapy for TNBC.

## Graphical abstract

Triple‐negative breast cancer still remains a major therapeutic challenge. Efforts are underway to utilize immunotherapies in clinical practice, but not all patients benefit. Comprehensive roles of hypoxia and immune status are rarely explored. Here we construct an index from crucial signatures, indicating the relative hypoxia‐immune status. Proposed treatments might include combined hypoxia modifiers and immunotherapies.

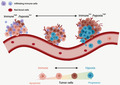

**Abbreviations**
AUC: area under the curve
BLIS: basal‐like immune‐suppressed
CA9: carbonic anhydrase 9
CA‐IX: carbonic anhydrase IX
CIBERSORT: Cell‐type Identification By Estimating Relative Subsets Of RNA Transcripts
CIHI: comprehensive index of hypoxia and immune
DCA: decision curve analysis
DFS: disease‐free survival
DMFS: distant metastasis‐free survival
ECM: extracellular matrix
EMT: epithelial–mesenchymal transition
FFPE: formalin‐fixed paraffin‐embedded
FISH: fluorescence in situ hybridization
GEO: Gene Expression Omnibus
Her2: human epidermal growth factor receptor 2
HR: hazard ratio
HRGs: hypoxia‐related genes
IF: immunofluorescence
IHC: immunohistochemistry
IM: immunomodulatory
IRGs: immune‐related genes
LAR: luminal androgen receptor
LASSO: least absolute shrinkage and selection operator
MATH: mutant‐allele tumor heterogeneity
MDSCs: myeloid‐derived suppressor cells
MES: mesenchymal‐like
METABRIC: Molecular Taxonomy of Breast Cancer International Consortium
MSigDB: Molecular Signature Database
OS: overall survival
pCR: pathologic complete response
PD‐L1: programmed death‐ligand 1
PFS: progression‐free survival
qRT‐PCR: quantitative real‐time PCR
ROC: receiver operator characteristic
ROS: reactive oxygen species
SRA: Sequence Read Archive
SYSUCC: Sun Yat‐sen University Cancer Center
TAMs: tumor‐associated macrophages
TCGA: The Cancer Genome Atlas
TMB: tumor mutation burden
TME: tumor microenvironment
TNBC: triple‐negative breast cancer

## 1 Introduction

Breast cancer is the most common cancer in women worldwide, with an estimated annual death of 41760 cases in women [1, 2]. Triple‐negative breast cancer (TNBC), a special subtype that represents 10–20% of patients with breast cancer, exhibits the most malignant biological behaviors and worst clinical outcomes [3]. Surgery and chemotherapy are considered the first‐line regimens for TNBC. Neither targeted therapy for Her2 (human epidermal growth factor receptor 2) nor endocrine therapy is applied for patients with TNBC in clinical practice [4]. The treatment for TNBC is so limited that it is urgent to develop effective therapies.

In previous studies, TNBC has been clustered into six different molecular subtypes according to genome‐wide profiling [5]. Lehmann et al. classified patients with TNBC into immunomodulatory (IM), basal‐like 1 (BL1), basal‐like 2 (BL2), mesenchymal (M), luminal androgen receptor (LAR), and mesenchymal stem‐like (MSL) groups. Moreover, Bareche et al. [6] found a higher level of immune features, including checkpoint molecules in the IM subtype. These results indicated that immunotherapies might be effective in particular subtypes of TNBC. Recently, several clinical trials have addressed the effect of immunotherapies on both early‐stage and metastatic TNBC, and there have been advances in our understanding of the biological and immunological characteristics of TNBC [7, 8]. Furthermore, the addition of immune checkpoint inhibitors could increase the pathologic complete response (pCR) rate of neoadjuvant chemotherapy in early‐stage TNBC [9]. Hence, immunotherapies have become novel promising candidates for TNBC treatment, and reliable indicators for treatment effect evaluation are indispensable. Although many different prognostic biomarkers and models have been developed to describe and quantify the immunological characteristics of TNBC, few have considered the effect of the extracellular microenvironment, such as hypoxia and pH, on cancer cells [10, 11].

The hypoxia‐related mechanism has long been considered one of the hallmarks in the cancer signaling pathway [12, 13, 14]. Hypoxia is a typical event in solid tumors and is associated with cancer metabolic reprogramming, stem cell signatures, angiogenesis, extracellular matrix (ECM) organization, and cancer cell metastasis [15, 16, 17]. Recent studies revealed novel roles of hypoxia in tumor progression and metastasis, including the induction of rapid methylation changes to histone and chromatin reprogramming, the development of an reactive oxygen species (ROS)‐resistant phenotype, and the induction of epithelial–mesenchymal transition (EMT) through macrophages [18, 19, 20].

Previous studies have investigated the association between hypoxia and the tumor microenvironment (TME). The hypoxia response in T lymphocytes enhances the expression of CD137 for immunotherapy [21]. The aerobic glycolysis of breast cancer cells could be regulated by lncRNAs from macrophages in the microenvironment [22]. Moreover, under hypoxic conditions, the dampening of NRF1 degradation could impair tumor‐associated macrophages (TAM) polarization [23]. However, hypoxia also drives CD8 + T‐cell migration and effector function, which indicates that hypoxia plays different roles in immune cells and tumor cells [24]. Although there are explicit links between hypoxia and the immune microenvironment, few studies have focused on the comprehensive status of hypoxia and the immune response in the TME.

Here, we aimed to develop and validate a comprehensive index of hypoxia and immune (CIHI) to reflect and quantify the microenvironment of TNBC based on these results. In this study, hypoxia‐related genes (HRGs) and immune‐related genes (IRGs) were screened for prognostic relevance and applied to model construction *in silico*. We confirmed that the CIHI was significantly correlated with hypoxia and immune status using bioinformatics analysis. We further evaluated the CIHI in tissue samples using quantitative reverse transcription polymerase chain reaction (qRT‐PCR) and immunofluorescence (IF) to further facilitate clinical application.

## 2 Materials and Methods

### 2.1 Study population and data acquisition

**Fig. 1:**
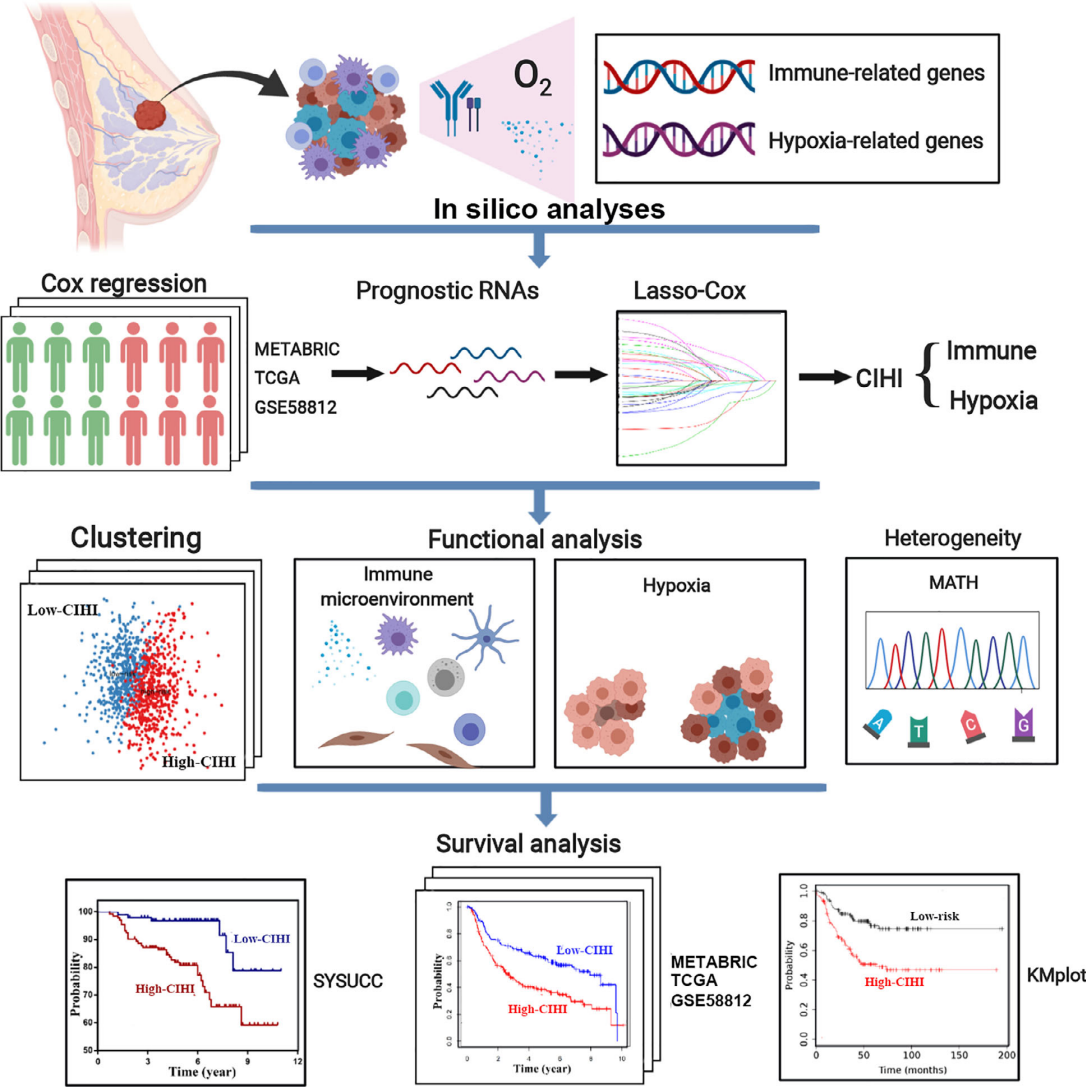
The work flow of this study. A panel of integrated prognostically relevant hypoxia‐related and immune‐related genes was identified in the METABRIC, TCGA, and GSE58812 datasets. A novel quantified index, the CIHI, was estimated using the LASSO Cox regression model, and its crucial roles in hypoxia and immune status were further validated in multiple cohorts.

The workflow is displayed in Fig. 1 and Fig. S1. For TNBC datasets from the Molecular Taxonomy of Breast Cancer International Consortium (METABRIC), The Cancer Genome Atlas (TCGA), and the Gene Expression Omnibus (GEO), patients who met the following selection criteria were included: (a) histologically diagnosed with malignant breast cancer; (b) available RNA expression data; and (c) available OS (overall survival) data. Patients without active follow‐up were excluded. In this study, 297 patients from METABRIC, 158 patients from TCGA, and 107 patients from GSE58812 were included. For CIBERSORT analysis, 293 patients from METABRIC, 137 patients from TCGA, and 103 patients from GSE58812 were included after screening. For TNBC RNA sequencing data from FUSCCTNBC cohort, 360 patients were included without overall survival information.

The METABRIC data were obtained from cBioportal (http://www.cbioportal.org/) [25]. The FUSCCTNBC data were downloaded from the Sequence Read Archive (SRA) (SRP157974, https://www.ncbi.nlm.nih.gov/sra) [26]. In addition, the expression profiles of the TCGA cohort were downloaded from the TCGA data portal (https://portal.gdc.cancer.gov/repository). The ensemble IDs were mapped to gene symbols according to the annotation of Homo_sapiens.GRCh38.91.chr.gtf from the ENSEMBLE website. The ‘limma’ package in R was used for gene expression normalization using the scale method [27]. The average RNA expression was calculated for duplicates, and genes with low abundance were discarded.

The GSE58812 expression profile was based on the GPL570 platform, and the expression matrix was obtained from the GEO database (https://www.ncbi.nlm.nih.gov/geo/) [28]. The probes were mapped according to the GPL570 annotation file (https://www.ncbi.nlm.nih.gov/geo/query/acc.cgi?acc=GPL570), and the average RNA expression was calculated for duplicates.

We complied with the access policies of the TCGA, METABRIC, SRA, and GEO databases in this study.

For the Sun Yat‐sen University Cancer Center (SYSUCC) cohort, the inclusion criteria were as follows: (a) histologically diagnosed as malignant breast cancer; (b) samples were available after surgery; (c) the molecular subtypes were confirmed by immunohistochemistry (IHC), and Her2 status was further validated using fluorescence in situ hybridization (FISH) if indecipherable in IHC; (d) AJCC 7^th^ TNM stage; (e) free from any other malignant tumors; and (f) no immunotherapies administered. Patients without active follow‐up records were excluded. Thirty patients with triple‐negative breast cancer between 2008 and 2011 were included in the SYSUCC study cohort. Overall survival was calculated from the date of diagnosis to the date of the last follow‐up or death. Disease‐free survival (DFS) was defined as the time from the date of diagnosis to the date of the first recurrence, including local recurrence and distant metastasis. Written informed consent was required for all patients, and this study was approved by the institutional review board of Sun Yat‐sen University Cancer Center and followed the guidance of the Declaration of Helsinki.

### 2.2 Generation of hypoxia and immune profiling

The HRGs were extracted from the hallmark gene sets in the Molecular Signature Database (MSigDB) (https://www.gsea-msigdb.org/gsea/msigdb/), and IRGs were obtained from ImmPort (https://www.immport.org). The available HRGs and IRGs in METABRIC, TCGA, and GSE58812 were included in this study. The functional protein–protein interaction network analysis was conducted by STRING (v11.0) (https://string-db.org). The position in chromosomes, expression, and interactions of these genes were exhibited in Circos plots by the ‘circlize’ package in r [29]. In total, 200 HRGs and 1811 IRGs were identified in all these cohorts and analyzed by univariate Cox regression for prognostic relevance. For analysis of previously reported hypoxia scores, the scores of the TCGA cohort were obtained from previous studies [30, 31, 32, 33, 34].

### 2.3 Development of the CIHI

The prognostically relevant HRGs and IRGs were identified using univariate Cox regression. The least absolute shrinkage and selection operator (LASSO) Cox regression model was further applied to determine the crucial signatures and the corresponding coefficients for model construction [35]. LASSO Cox regression was conducted using the ‘glmnet’ package in R software, and the ideal coefficients were estimated according to the partial likelihood deviance with ten‐fold cross validation [36]. The optimal log λ was determined at −3.37. To quantify the comprehensive effects of hypoxia and immune status, a novel score was calculated from the signatures selected by the LASSO model. The following formula was used:

$$\text{Score}\;=\;\sum_{i=1}^{n}\text{(Genei}\times \text{Coefi)}$$

For clinical utility, the comparative value (2^−ΔCt^) was calculated from qRT‐PCR results and used for score calculation in the SYSUCC cohort. The score was further standardized and simplified to generate a comprehensive index of hypoxia and immune. The score was subsequently mapped by subtracting the minimum and dividing by the maximum. Mapping was conducted to facilitate the interpretation of results from different platforms. The CIHI was calculated as follows:

$$\text{CIHI}=\left(\text{Score}-\text{Min}\right)/\text{Max}.$$

### 2.4 Tumor microenvironment analysis

For Cell‐type Identification By Estimating Relative Subsets Of RNA Transcripts (CIBERSORT) analysis, the expression matrices were uploaded to the online analytical platform (https://cibersort.stanford.edu), and the proportions of infiltrating immune cells were estimated according to LM22 signatures with 1000 permutations [37]. The qualified samples were then selected using a criterion of *P* < 0.05. xCell analysis was performed according to the guidance of the website https://xcell.ucsf.edu [38]. Immune and stromal scores were further estimated to quantify the immune and stromal components by the ESTIMATE algorithm using the ‘estimate’ package in R [39]. MCP‐counter scores regarding immune‐related activity and fibroblasts were evaluated using the ‘MCPcounter’ package in R [40]. ssGSEA was performed to calculate the enrichment score of specific immune signatures in samples using the ‘GSVA’ package in R according to a previous study [41]. For comparison of the hypoxia and immune status between the low‐risk and high‐risk groups, the key expression profiles were compared.

### 2.5 Functional study and heterogeneity

The GSEA was conducted to explore the pathway enrichment between low‐risk and high‐risk groups using GSEA 3.0. The annotated gene set was downloaded for reference (http://software.broadinstitue.org). The tumor mutation burden (TMB) and the mutant‐allele tumor heterogeneity score (MATH) for the TCGA cohort were estimated by the ‘maftools’ package in R software [42]. PCA was used to examine the clustering efficacy of the selected signatures in the LASSO model.

### 2.6 RNA isolation and quantitative real‐time PCR

The total RNA of breast cancer tissues was extracted using TRIzol reagent (Invitrogen, Carlsbad, California, USA). Reverse transcription was performed according to the manufacturer’s instructions (Takara, Kusatsu, Japan). The expression levels of the target genes were further examined in triplicate using the SYBR Green method (Takara). The primers involved in this study are provided in Table S1. The expression levels were normalized to that of β‐actin with the comparative Ct method.

### 2.7 Immunofluorescence

Immunofluorescence (IF) was performed as previously described [43]. The formalin‐fixed paraffin‐embedded (FFPE) sections were deparaffinized, and we performed the antigen‐retrieval procedure at 98°C in citrate buffer (pH 6.0) for 10 min. The endogenous peroxidase blocking procedure was then carried out using 3% hydrogen peroxide for 10 min at room temperature. The sections were further incubated with anti‐carbonic anhydrase 9 (CA9) (Proteintech, Chicago, Illinois, USA) or programmed death‐ligand 1 (PD‐L1) (Proteintech) at 4°C overnight. IgG (CST, Danvers, Massachusetts, USA) was used as a negative control. Then, the samples were washed with PBS three times and incubated with anti‐mouse or anti‐rabbit secondary antibodies, namely, Alexa Fluor‐594 or Alexa Fluor‐488 (1 : 1000, Invitrogen). A Zeiss LSM 880 confocal microscope was used to observe the results, and images were acquired. The reagents are shown in Table S2.

### 2.8 Statistical analysis

Univariate Cox regression was used to identify the prognostically relevant HRGs and IRGs in the METABRIC, TCGA, and GSE58812 cohorts with a cutoff value of *P* < 0.1. Crucial signatures involved in hypoxia and immune status were identified by the LASSO Cox regression model. The multivariate Cox regression model was constructed using the ‘survival’ package to include the CIHI and clinical predictors. The optimal cutoff value of survival analysis was determined using the ‘survminer’ package in R, and the OS and DFS of different subgroups were compared using the Kaplan–Meier method with the log‐rank test. Time‐dependent receiver operator characteristic (ROC) analyses were performed using the ‘timeROC’ package in R [44]. Decision curve analysis (DCA) was performed in R software using a previously reported method [45]. The significance of the difference between immune cell fractions was assessed by the Wilcoxon test. Spearman’s correlation test was used for CIHI‐related analysis. Statistical analyses were performed using R software (Version 3.6.0). A *P* value of < 0.05 was considered statistically significant, and all *P* values were two‐tailed.

## 3 Results

### 3.1 Overview of hypoxia and immune signatures

**Fig. 2:**
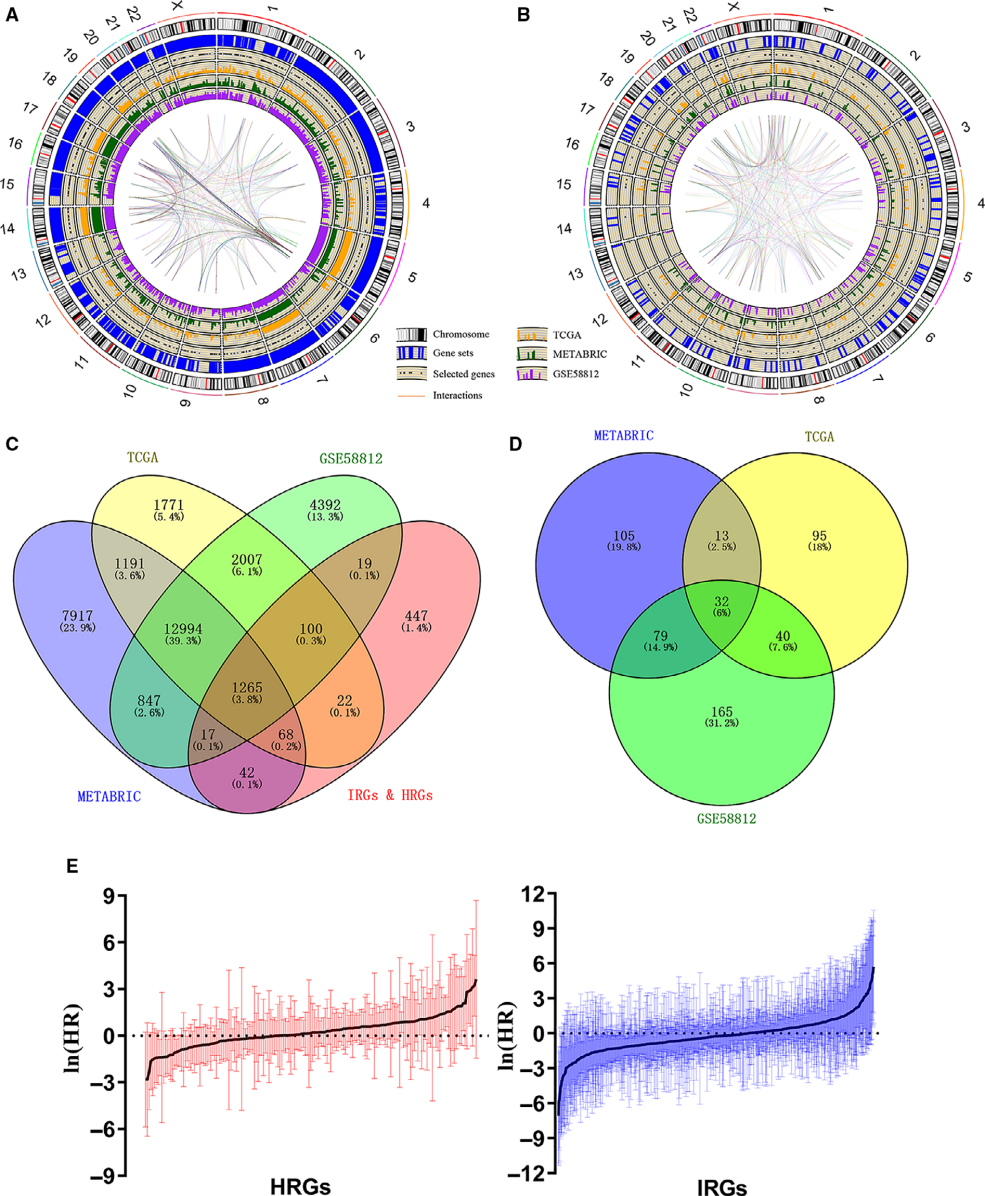
Identification of prognostic HRGs and IRGs in TNBC. (A, B) Circos plots of the annotation and interaction of HRGs and IRGs in the genome. The outer circle shows the positions of individual genes on chromosomes. The scatters in the second circle represent the identified genes in all three cohorts. The third to fifth circles indicate the relative expression levels of the identified genes in the METABRIC, TCGA, and GSE58812 cohorts. The central lines represent the potential interactions between genes predicted by the STRING database. (C) Venn diagram indicating 1265 genes identified in all three cohorts. (D) Venn diagram indicating 32 prognostic genes identified in all three cohorts. (E) Barplot showing the hazard ratio of the HRGs and IRGs in the METABRIC cohort. The bars represent the *95% CI*. Univariate Cox regression was used for data analysis.

A population of 297 patients from METABRIC, 158 patients from TCGA, and 107 patients from GSE58812 were identified and included in this study. The transcriptome data were used to construct a comprehensive indicator from hypoxia and immune profiling (Fig. 2A,B). Two hundred hypoxia‐related genes and 1811 immune‐related genes were used for model construction. A total of 715 genes were not identified in all datasets and were excluded from the survival analysis (Fig. 2C). A univariate Cox regression model was applied to determine the prognostic relevance of these signatures, and 32 prognostically relevant genes in the METABRIC, TCGA, and GSE58812 cohorts were identified (Fig. 2D,E).

### 3.2 Construction of a comprehensive index of hypoxia and immune status in TNBC

**Fig. 3:**
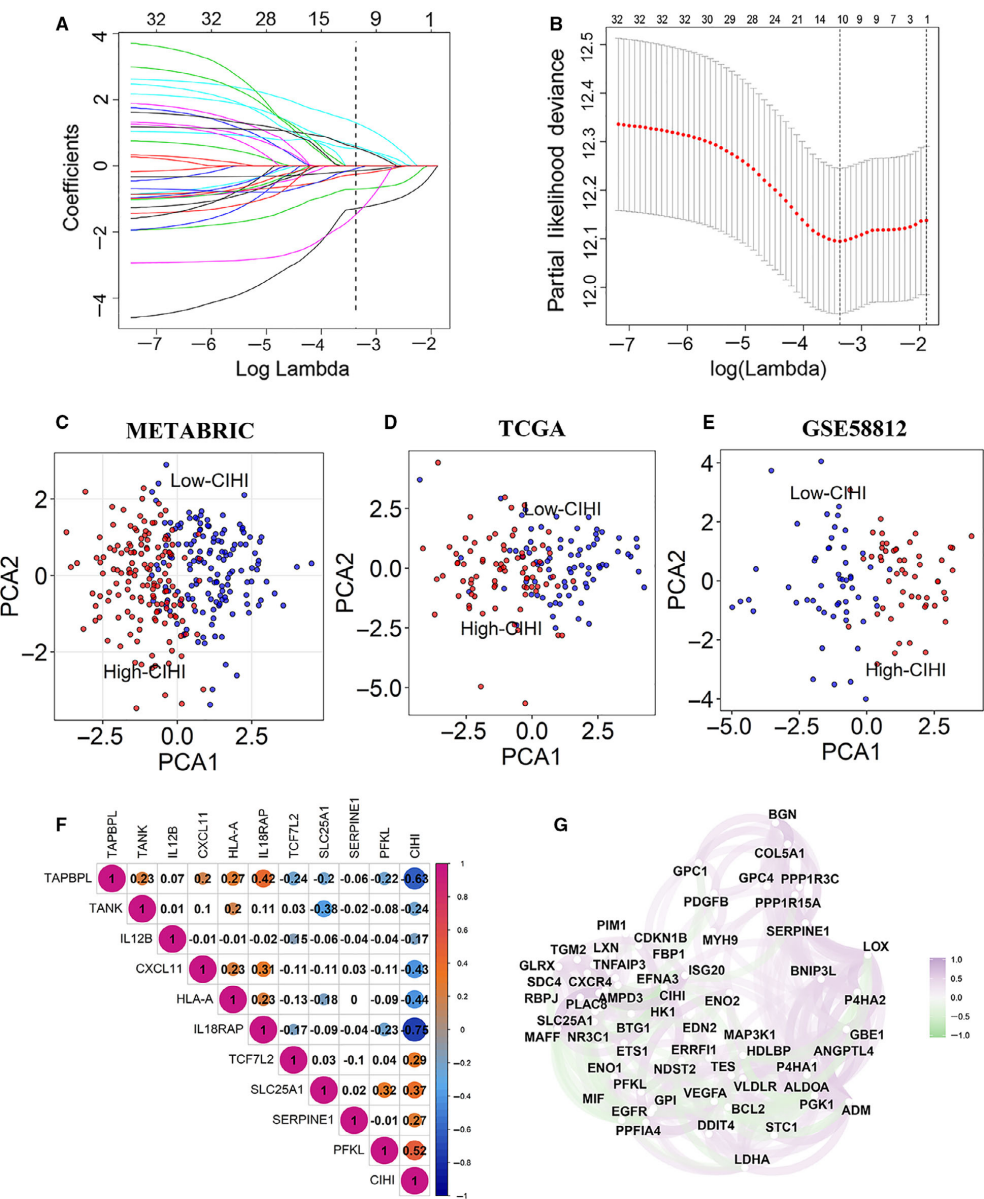
Construction of a predictive model and the CIHI. (A, B) The LASSO Cox regression model was constructed from 32 signature genes, and the tuning parameter (λ) was calculated based on the partial likelihood deviance with ten‐fold cross validation. An optimal log λ value was defined at −3.37, as shown by the vertical black lines in the plots. The ten signature genes were identified according to the best fit profile. (C–E) PCA based on the expression profile of the 10 selected signature genes according to different risk groups. (F) Correlation between the CIHI and the selected signature genes in the METABRIC cohort. (G) Correlation network between the CIHI and its correlated hypoxia‐related genes in the METABRIC cohort. Data were analyzed using Spearman’s rank correlation analysis.

Given that a hypoxic microenvironment might affect the activation status of infiltrating immune cells and the immune response of tumor cells, an integrated analysis of both hypoxia and immune response might have potential prognostic value and quantify the TME. Hence, 32 prognostically relevant signature genes were applied to the LASSO Cox regression model to construct a predictive model for the overall survival of patients with TNBC in the METABRIC dataset (*N* = 297). Ten genes were selected according to the partial likelihood deviance method, and the corresponding coefficients were generated at the optimal log λ of −3.37. The results are shown in Fig. 3A,B. Among the 10 signature genes, PFKL, SLC25A1, and SERPINE1 were hypoxia‐related genes, while the others were immune‐related genes (TAPBPL, CXCL11, HLA‐A, TCF7L2, TANK, IL12B, and IL18RAP). Kaplan–Meier analysis further confirmed the prognostic value of the individual genes (Fig. S2). The selected signature genes were applied to the formula above, and the CIHI was calculated. Moreover, the CIHI was also estimated in the TCGA and GSE58812 cohorts using this method.

To further facilitate the application of the CIHI in TNBC, the patients were divided into low‐risk and high‐risk groups according to the median value of the CIHI. PCA showed that patients in different groups could be distinctively clustered according to the selected signatures in all datasets (Fig. 3C–E). In addition, Spearman’s correlation test indicated that the standardized CIHI was significantly correlated with the selected genes. The correlations among these signatures are shown in Fig. 3F.

Previous studies suggested that a high level of hypoxia could lead to immune suppression in the TME [46, 47]. These results demonstrated that hypoxia was associated with the immune response in the microenvironment. Thus, this novel scoring method, the CIHI, might be indicative of the hypoxia–immune status of patients and has importance in clinical guidance.

### 3.3 Validation of hypoxia profiling in CIHI

The analysis above was used to construct a comprehensive indicator and identify two distinctive subgroups of patients with TNBC. Correlation analysis in the METABRIC cohort showed a correlation between the CIHI and hypoxia‐related genes, indicating that the CIHI might reflect hypoxia in the TME (Fig. 3G). Hence, we further sought to validate the correlation of the CIHI and hypoxia. A previous study addressed the key hypoxia‐related expression profiles in cancer [48]. We first compared the key hypoxia‐related signatures in the high‐risk and low‐risk groups.

**Fig. 4:**
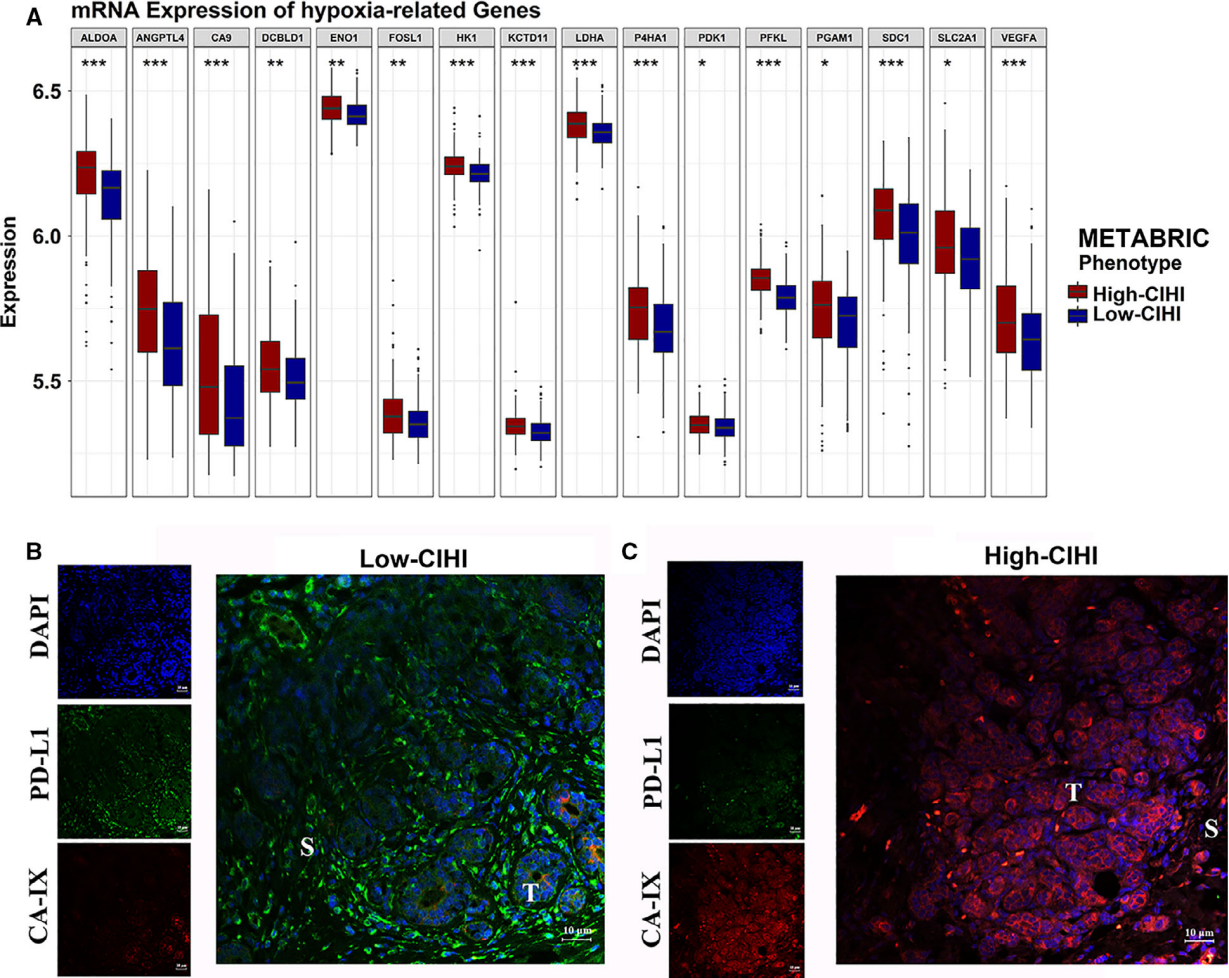
Hypoxia‐related profiling and immunofluorescence in the CIHI‐based groups. (A) Box and whisker plots showing the expression of the selected hypoxia‐related genes in the METABRIC cohort. The high‐CIHI and low‐CIHI phenotypes are represented as dark red and dark blue, respectively. Student’s *t*‐test was used for data analysis. (B, C) Representative images of hypoxia and PD‐L1 in the tumor microenvironment of the CIHI‐based subgroup in the SYSUCC cohort using immunofluorescence. PD‐L1 protein in stroma is shown in green color, and red color in tumor cells indicates CA‐IX protein. T, tumor; S, stroma. *P* values are shown as *P < 0.05, **P < 0.01, and ***P < 0.001.

Subsequent analyses mapped the expression level of genes in the two phenotypes of the METABRIC cohort to reflect the distinct hypoxia status. Sixteen genes showed a statistically significant difference between the subgroups (low‐risk group < high‐risk group). The expression of ALDOA, ANGPTL4, CA9, DCBLD1, ENO1, FOSL1, HK1, KCTD11, LDHA, P4HA1, PDK1, PFKL, PGAM1, SDC1, and VEGFA was significantly higher in the high‐risk group (Fig. 4A). The results showed that hypoxia‐induced metabolic rearrangement and angiogenesis were more common in the high‐risk group.

To further validate the results, qRT‐PCR was performed in the SYSUCC cohort (*N* = 30) to facilitate the clinical application of the CIHI. Based on the relative Ct values of the ten selected signature genes described above, the CIHI was estimated using our previous formula. Previous studies addressed the indicative function of Carbonic anhydrase IX (CA‐IX) in breast cancer and made it a reliable reflection of hypoxia [49, 50, 51]. Therefore, the protein level of CA‐IX was examined in these patients using IF (Fig. 4B,C). Patients with high CIHI values were prone to exhibit a higher level of CA‐IX protein in tumor cells. We also detected the PD‐L1 protein because PD‐L1 is a crucial target for immunotherapy and reflects a potential immune response in tumors. Interestingly, patients with low CIHI values exhibited a lower level of hypoxia but a higher level of PD‐L1, which was consistent with the following results regarding immune status. Moreover, a stromal PD‐L1 expression was more prevalent. Hence, the low‐risk and high‐risk groups were more likely to exhibit hypoxia^low^/immune^high^ and hypoxia^high^/immune^low^ phenotypes, respectively.

### 3.4 Immune profiling

**Fig. 5:**
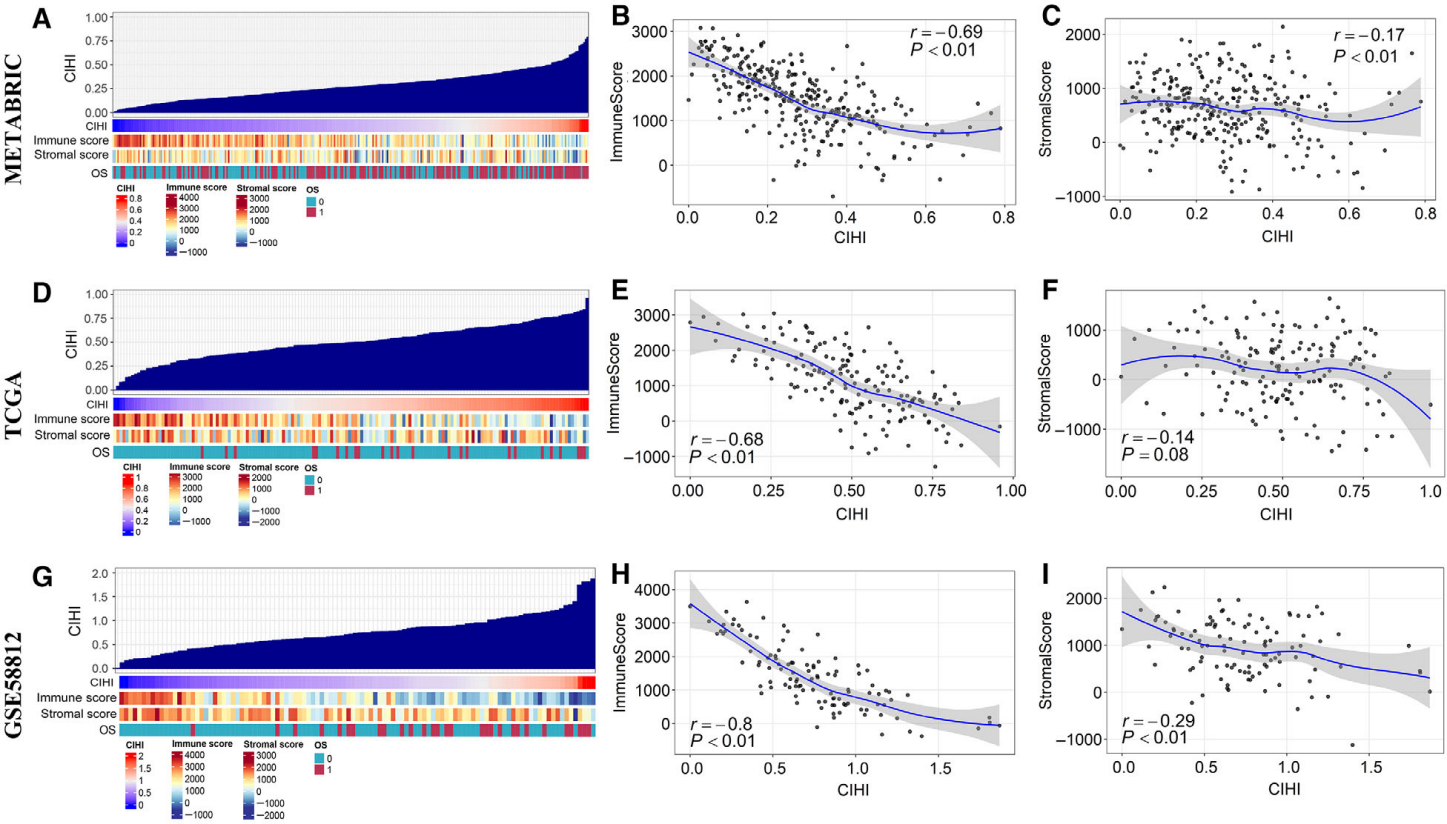
Potential indicative value of the CIHI in the TME and clinical outcomes. (A, D, G) An overview of the association between OS event, stromal score, immune score, and the CIHI in TNBC patients from three independent cohorts. Columns represent samples sorted by the CIHI from low to high. Rows represent the coefficients of interest. (B, E, H) Correlation between the CIHI and immune score in the cohorts. (C, F, I) Correlation between the CIHI and the stromal score in the cohorts. Data were analyzed using Spearman’s rank correlation analysis.

Further results showed a continuity feature of the CIHI and its correlation with the TME and clinical prognosis in the METABRIC, TCGA, and GSE58812 cohorts (Fig. 5A,D and G). The value of the CIHI was mapped into a continuous scale, which indicated potential clinical relevance in predicting the prognosis of patients. For overall survival, patients with higher CIHI values seemed to have a higher rate of death. Moreover, the ESTIMATE method was employed to explore the overall TME status. A low immune score and stromal score were more commonly observed in patients with a higher CIHI. Further analysis indicated a significant inverse correlation between the CIHI and immune score in the METABRIC (*r *= −0.69, *P* < 0.01), TCGA (*r *= −0.68, *P* < 0.01) and GSE58812 (*r *= −0.80, *P* < 0.01) cohorts (Fig. 5B,E and H). Interestingly, the CIHI was also negatively correlated with the stromal score, indicating that the CIHI might also be associated with stromal nonimmune components, such as fibroblasts (Fig. 5C,F and I).

**Fig. 6:**
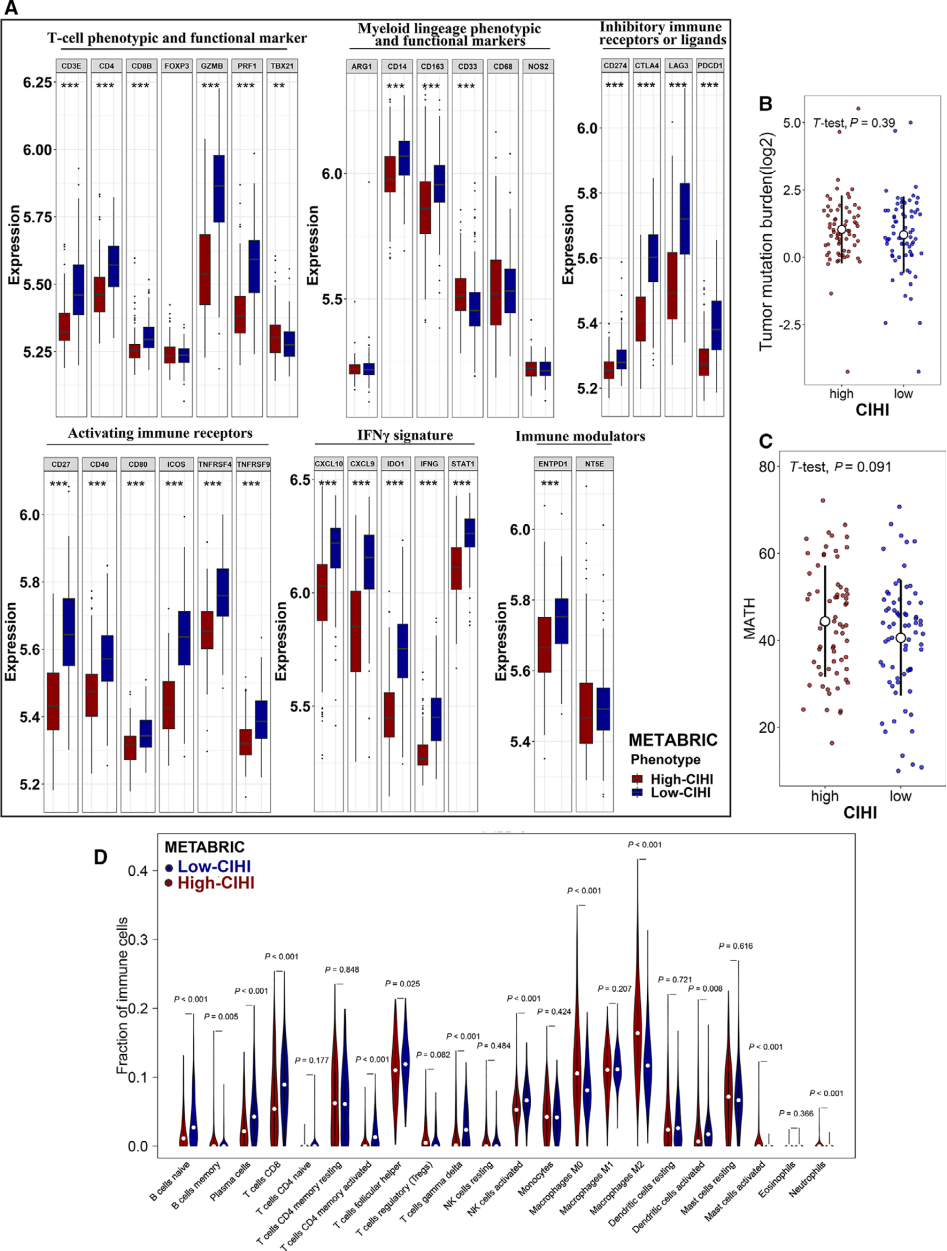
Immune‐related profiling, tumor‐infiltrating immune cells, and genomic heterogeneity in the CIHI‐based groups. (A) Box and whisker plots show the expression of selected immune‐related genes in the METABRIC cohort. (B, C) TMB and the MATH score in the high‐CIHI and low‐CIHI groups. (D) Comparison of infiltrating immune cells (CIBERSORT) between groups. The hypoxia^high^/immune^low^ and hypoxia^low^/immune^high^ phenotypes are represented as dark red and dark blue, respectively. Student’s t‐test and Wilcox test were used for data analysis. *P* values are shown as **P < 0.01 and ***P < 0.001.

To further facilitate the indicative function of the CIHI in the immune response, a panel of immune‐response signatures was also mapped into the hypoxia^high^/immune^low^ and hypoxia^low^/immune^high^ phenotypes (Fig. 6A). Immune response‐related signatures were mostly differentially expressed: hypoxia^high^/immune^low^ < hypoxia^low^/immune^high^. Our results demonstrated a different pattern of immune response between the phenotypes. Overall, patients with the hypoxia^high^/immune^low^ phenotype exhibited a suppressive immune microenvironment compared with patients with the hypoxia^low^/immune^high^ phenotype. This finding was consistent with the fact that these signatures were responsible for mediating pro‐ or antitumoral activity. The phenotypic and functional markers of T cells, namely, CD3E, CD4, CD8B, GZMB, PRF1 and TBX21, were expressed at higher levels in the hypoxia^low^/immune^high^ phenotype, although FOXP3 did not show a significant difference. For the myeloid lineage phenotypic and functional markers, CD14 and CD163 were highly expressed in the hypoxia^low^/immune^high^ group, indicating a higher percentage of monocytes and M2 macrophages. However, CD33, a previously reported marker for M‐MDSCs, was highly expressed in the hypoxia^high^/immune^low^ group. Other markers, including IFNγ signatures (CXCL10, CXCL9, IDO1, IFNG, and STAT1) and immune modulators (ENTPD1), also exhibited a higher expression level in the low‐risk group (hypoxia^low^/immune^high^). Interestingly, a higher level of inhibitory immune receptors or ligands (PD‐L1, CTLA4, LAG3, and PD‐1) and activating immune receptors (CD27, CD40, CD80, ICOS, TNFRSF4, and TNFRSF9) were both observed in the hypoxia^low^/immune^high^ phenotype, indicating a complex immune response in the low‐risk group.

Then, we analyzed the tumor mutation burden (TMB) and tumor heterogeneity based on genome‐wide methods. Although a distinct immune response was observed in different phenotypes, the TMB did not show a consistent result (Fig. 6B). However, the MATH score, a quantified scale for genome heterogeneity, demonstrated a higher level of tumor heterogeneity in the high‐risk group (*P* = 0.091) (Fig. 6C). This result was consistent with that of a previous study showing that the hypoxic microenvironment might exert an effect on the genome.

### 3.5 The relevance of the CIHI and hypoxia–immune markers

As described above, qRT‐PCR of the ten‐gene signature was performed, and the CIHI was calculated for patients in the SYSUCC cohort. The hypoxia and immune markers were then examined in tumor samples by IF. CA‐IX was previously reported to be a reliable marker for hypoxia in breast cancer and was mainly expressed in cancer cells instead of stromal cells. A high level of CA‐IX often indicates a hypoxic microenvironment in tissues. PD‐L1 is widely recognized as a potential immune marker and therapeutic target for cancers. Patients with a higher CIHI were more likely to exhibit positive CA‐IX and negative stromal PD‐L1 expression (Fig. 4B,C). However, due to the limited number of patients, we did not find any convincing evidence for the correlation between CA‐IX and tumoral PD‐L1 expression.

To further facilitate our hypothesis in larger cohorts, we first estimated the immune cell fractions in the METABRIC cohort using the CIBERSORT algorithm (Fig. 6D). Patients in the low‐risk group exhibited a higher percentage of antitumoral immune cells, including naive B cells (*P* < 0.01), CD8 + T cells (*P* < 0.01), activated memory CD4 + T cells (*P* < 0.01), activated NK cells (*P* < 0.01), and activated dendritic cells (*P* < 0.01), while patients in the high‐risk group showed a higher level of regulatory T cells (*P = *0.08) and M2 macrophages (*P* < 0.01). Moreover, the CIBERSORT results for the TCGA and GSE58812 cohorts are shown in Fig. S3. The high‐risk group presented with a higher percentage of M1 macrophages and a lower percentage of M2 macrophages in both cohorts. Infiltrating cell scores in TME were also estimated by xCell, and similar results were shown in Table S3.

**Fig. 7:**
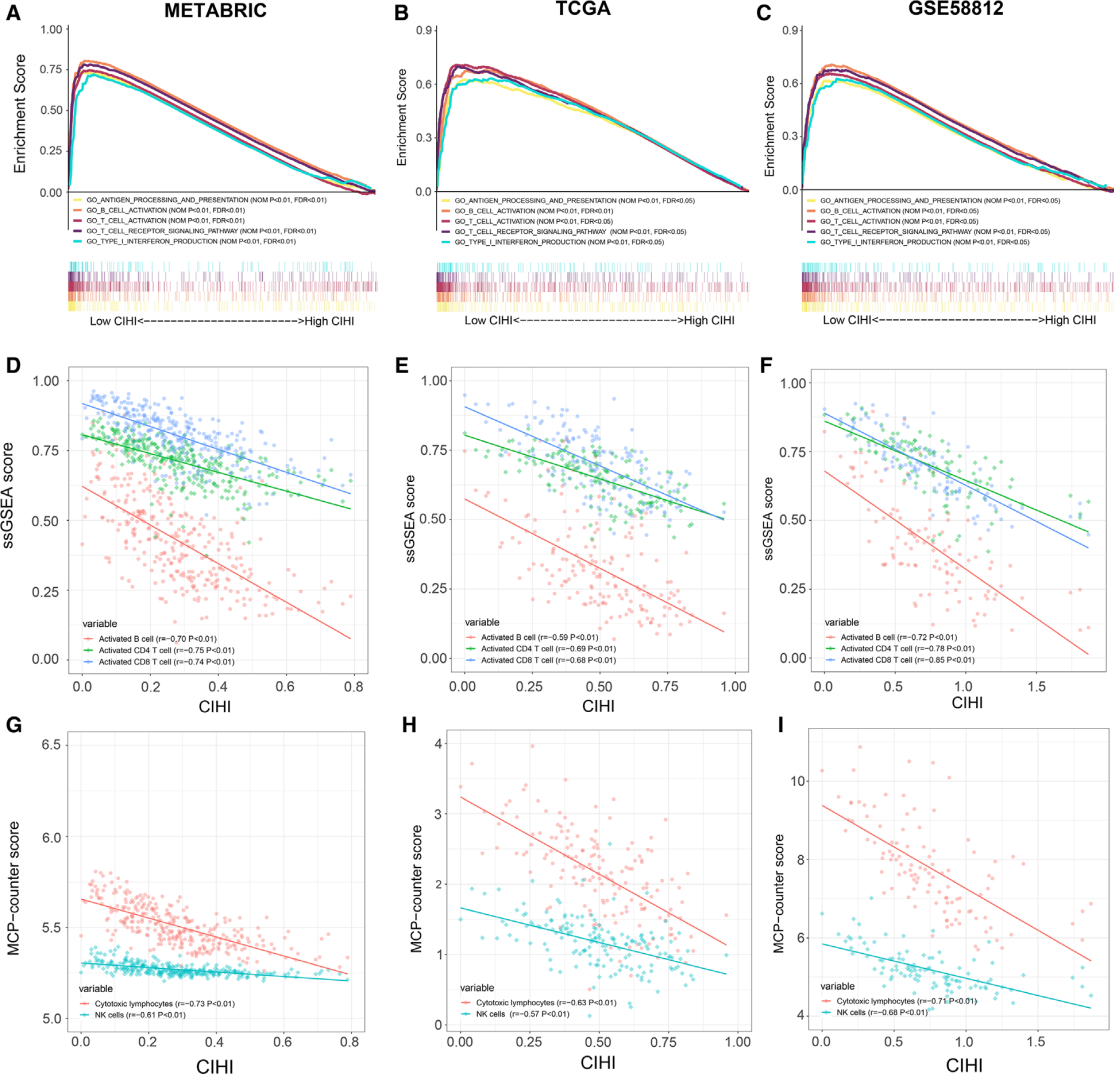
Associated immune processes and cells with the CIHI. (A–C) GSEA of enriched immune‐related signaling in the CIHI‐based groups. (D–F) Associated ssGSEA‐estimated immune cell activation with the CIHI. (G–I) Associated MCP‐counter‐estimated infiltrating cells with the CIHI. Data were analyzed using Spearman’s rank correlation analysis.

We then applied GSEA to examine the relevant signaling pathways involved in patients with a low CIHI. Our results demonstrated that antigen processing and presentation, B‐cell activation, and T‐cell activation were significantly enriched in the hypoxia^low^/immune^high^ group (Fig. 7A–C). Furthermore, more algorithms were employed to validate the association between the CIHI and infiltrating immune cells. Using a previously reported ssGSEA method, the crucial signals regarding immune cells were estimated and scored in the METABRIC, TCGA, and GSE58812 cohorts. Significant reverse correlations between the CIHI and activated B cells (*r *= −0.70, *P* < 0.01), activated CD4 + T cells (*r *= −0.75, *P* < 0.01), and activated CD8 + T cells (*r *= −0.74, *P* < 0.01) were observed in the METABRIC cohort (Fig. 7D). The results were further validated in the TCGA and GSE58812 cohorts (Fig. 7E,F). The MCP‐counter score demonstrated that the CIHI was inversely correlated with cytotoxic lymphocytes and NK cells in these cohorts (Fig. 7G–I).

### 3.6 Prognostic value of the CIHI

The development of convenient tools for the early diagnosis of diseases and treatment guidance remains a crucial clinical issue. Previous studies have demonstrated that hypoxia and immune status are indicators of malignant tumors. In our study, as shown above, the CIHI exhibited a continuity feature, and remarkably heterogeneous characteristics of hypoxia and immune status were observed between the hypoxia^high^/immune^low^ and hypoxia^low^/immune^high^ phenotypes. More death events were observed in patients with higher CIHI values. To further elucidate the predictive and prognostic value of the CIHI in TNBC, ROC analysis and the Kaplan–Meier method were used to examine and validate prognosis in the cohorts.

**Fig. 8:**
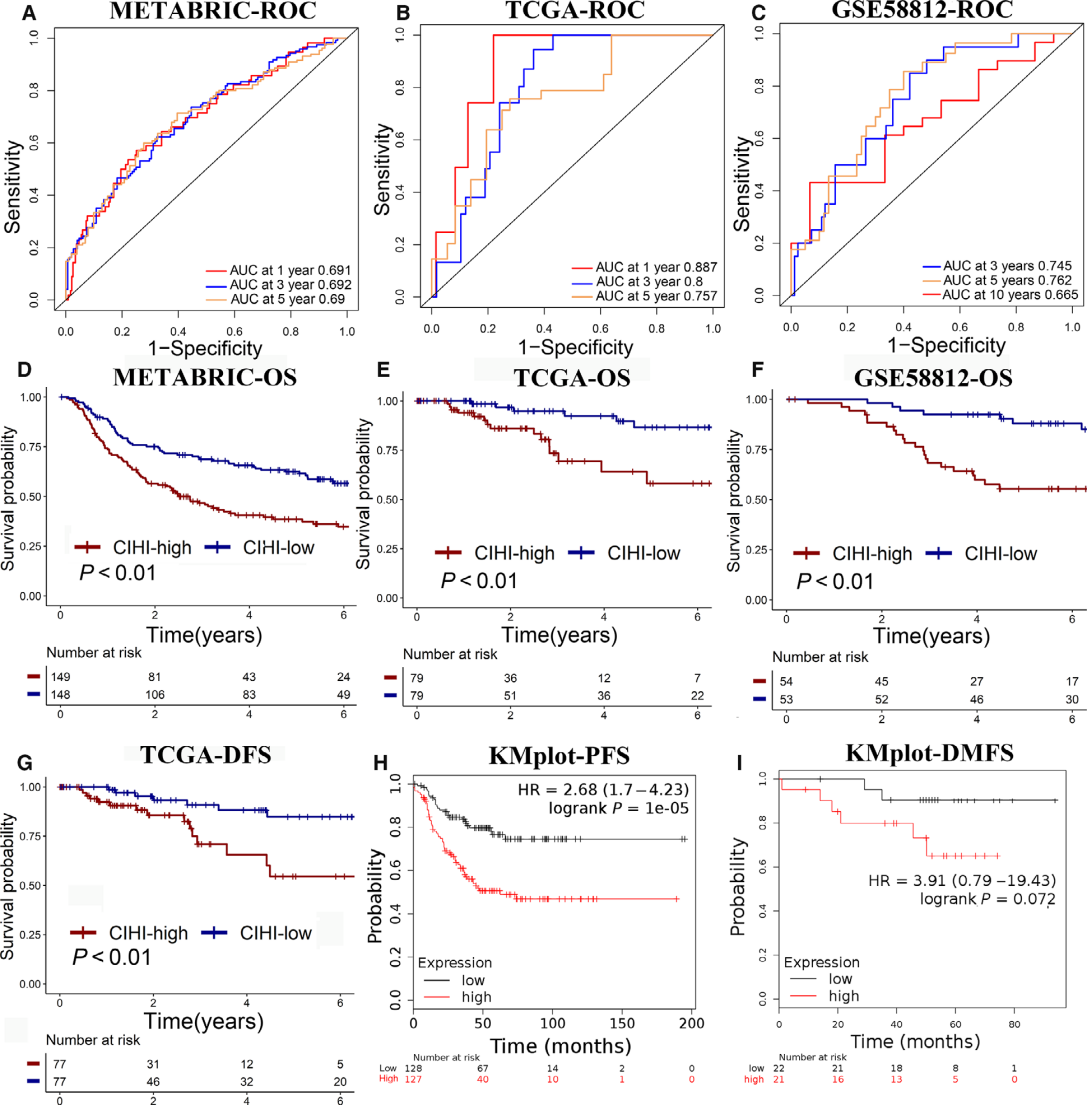
(A–C) Time‐dependent ROC analysis of the CIHI regarding OS in patients with TNBC. (D–F) Kaplan–Meier plots of OS in the CIHI‐based groups. (G) Kaplan–Meier plot of DFS in the TCGA cohort. (H, I) Kaplan–Meier plot of PFS and DMFS in the KMplot database. Log‐rank test was used for data analysis.

Time‐dependent ROC analysis was performed, and the area under the curve (AUC) was calculated at different time points according to data availability (Fig. 8A–C). Generally, the ROC analysis of the 5‐year follow‐up in the METABRIC (AUC = 0.690), TCGA (AUC = 0.757), and GSE58812 (AUC = 0.762) cohorts indicated a favorable predictive value of the CIHI in long‐term follow‐up. Moreover, the CIHI seemed to present with an evidently better predictive ability for OS in the TCGA cohort during short‐term follow‐up (AUC = 0.887). These results indicated that the CIHI could serve as a clinical biomarker. The decision curve analysis also confirmed the predictive value of the CIHI in cohorts (Fig. S4).

Overall survival was compared in patients with different CIHI values. The patients in the hypoxia^low^/immune^high^ group exhibited a better OS than those in the high‐risk group in the METABRIC cohort, the TCGA cohort, and the GSE58812 cohort (Fig. 8D–F). Moreover, patients with higher CIHI values also showed a poorer outcome for DFS in the TCGA cohort (Fig. 8G). The online database KM plotter facilitated the construction of predictive models and evaluation of prognosis in breast cancer patients. The risk score was calculated with the KM plotter, and progression‐free survival (PFS) and distant metastasis‐free survival (DMFS) were calculated as described above (Fig. 8H,I). Patients with a low risk score had a better PFS [hazard ratio (HR), 2.68; 95% confidence interval (95% CI), 1.7–4.23; *P* < 0.01] and DMFS (HR, 3.91; 95% CI, 0.79–19.43; *P* = 0.072). The CIHI was calculated for patients in the SYSUCC cohort, and the hypoxia^low^/immune^high^ group showed significantly better OS and DFS compared with the hypoxia^high^/immune^low^ group (Fig. S5).

We then performed univariate and multivariate Cox regression analyses in the METABRIC cohort to support the clinical relevance of the CIHI. In the univariate Cox regression model, the high‐risk group (HR, 1.699; 95% *CI*, 1.176–2.455; *P < *0.01), age > 65 years (HR, 1.710; 95% CI, 1.176–2.485; *P* < 0.01), larger tumor size (HR, 1.481; 95% *CI*, 1.118–1.962; *P* < 0.01), and TNM stage III‐IV (HR, 1.538; 95% *CI*, 1.130–2.095; *P* < 0.01) were risk factors for TNBC. However, in the multivariate Cox regression model, age> 65 years (HR, 1.675; 95% CI, 1.138–2.466; *P* < 0.01) and the high‐risk group (HR, 1.491; 95% CI, 1.018–2.183; *P* < 0.05) were identified as independent risk factors (Fig. S6). A larger tumor volume might result in the intratumor hypoxic status, and we analyzed the correlation between the CIHI and tumor size (Fig. S7). A positive correlation between the CIHI and tumor size was observed in the METABRIC dataset (*r* = 0.21, *P* < 0.01), and in the TCGA dataset, patients with T3‐4 stage were significantly characteristic of higher CIHI value (*P* = 0.019). However, more evidence and larger cohorts are necessary for further validation.

**Fig. 9:**
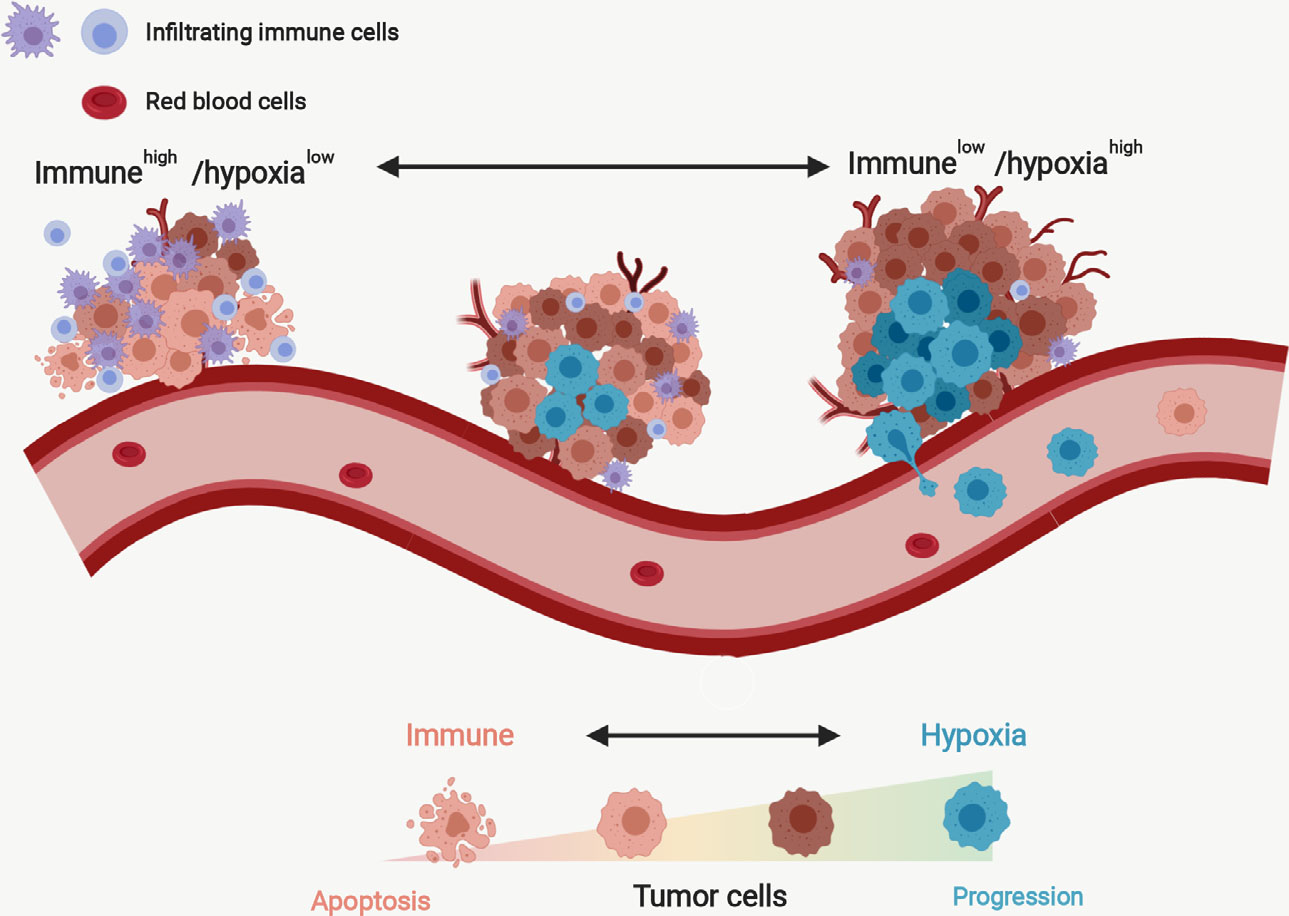
Proposed relatively indicative roles of the CIHI in the relative hypoxia and immune status of the tumor microenvironment. A lower CIHI predicted relatively active immune response, and a relatively higher CIHI was more likely to predict a hypoxia^high^/immune^low^ status.

Furthermore, we sought to validate the indicative role of the CIHI for hypoxia and immune characteristics in a larger cohort. The data from FUSCCTNBC cohort were analyzed, and patients were divided into groups according to the median value of CIHI. The hypoxia markers were highly expressed in the high‐CIHI group (Fig. S8a). The ESTIMATE immune and stromal scores were also inversely correlated with the CIHI (Fig. S8b,c). MCP‐counter and ssGSEA scores indicated that patients with lower CIHI value were characteristics of more tumor‐infiltrating cytotoxic immune cells (Fig. S8d,e). The CIHI value was compared among the previously reported subtypes, and patients with the IM (immunomodulatory) subtype showed a significantly lower CIHI value, which further supported our findings (Fig. S8f). Previously reported hypoxia scores were significantly higher in CIHI‐high group (Fig. S9). Therefore, a lower CIHI might predict relatively active immune response, and a relatively higher CIHI was more likely to predict a hypoxia^high^/immune^low^ status (Fig. 9).

## 4 Discussion

Previous studies have addressed the important role of hypoxia and immune status features in breast cancer [52, 53]. Recent studies have advanced our understanding of the efficacy of immunotherapies in TNBC, and promising results have emerged in several clinical trials [7, 8, 9]. Researchers have focused on the effect of targeted therapies on immune checkpoints in triple‐negative breast cancer, and the effect of the combination of PD‐L1/PD‐1 blockade with routine therapies has been investigated.

As shown in previous studies, the hypoxic microenvironment drives the suppression of immune status in breast cancer. The tumor microenvironment is reprogrammed under the pressure of hypoxia, resulting from the modulation of CD8 + cytotoxic T cells and macrophages [21, 23, 24, 46, 54]. Moreover, the metabolic reprogramming of tumor cells provides an adaptation under hypoxia by increasing lactate production and glucose uptake, contributing to the development of an immunosuppressive microenvironment [55, 56]. Interestingly, crosstalk from TAMs to breast cancer cells by exosomes regulates HIF‐1α‐mediated aerobic glycolysis metabolism and contributes to tumor progression in breast cancer [22].

Although clinical indicators regarding hypoxia or immune status have been developed, few studies have focused on their comprehensive effects and their potential roles in clinically relevant classification and therapy selection. Generally, patients with similar clinical characteristics still present with great heterogeneity in clinical outcomes, and integrated predictors from single biomarkers could effectively improve the prognostic value.

Given that hypoxia modifiers and immune checkpoint inhibitors have been shown to exhibit a potential effect on breast cancer, we explored the potential role of a combined hypoxia and immune status classifier for TNBC in this study [57, 58]. The use of a combined immune–hypoxia signature in a cross‐cohort manner helped to develop a continuous index for comprehensive TME assessment. Subgroup classification divided the population into the hypoxia^high^/immune^low^ and hypoxia^low^/immune^high^ groups, correlating with distinct clinical prognosis, transcriptional hypoxia–immune patterns, and activated pathways that could serve as therapeutic targets. Therefore, we addressed the importance of separating them.

The key markers of hypoxia (CA9, ALDOA, ENO1, LDHA, etc.) are highly expressed in the hypoxia^high^/immune^low^ group compared with the hypoxia^low^/immune^high^ group, indicating heterogeneous hypoxia statuses within the population. In addition, immune cell infiltration was also investigated to further validate our model. Infiltrating immune cells, including cytotoxic T cells, macrophages and NK cells, are widely recognized as crucial immune indicators in cancers [59, 60, 61]. In our study, a higher percentage of cytotoxic T cells, M2 macrophages, and NK cells were observed in the hypoxia^low^/immune^high^ group. PD‐L1 expression, especially within stromal cells in the TME, is correlated with clinical prognosis in breast cancer and may reflect an adaptive immune response. Previous studies have revealed the distinct role of PD‐L1 expression in stromal immune cells and tumor cells [62, 63]. The PD‐L1 protein is always positive in tumor cells when that in immune cells is positive [64]. Moreover, patients with positive PD‐L1 expression are more likely to exhibit better prognosis. Hence, it is more reasonable and convenient to use the stromal PD‐L1 as an indicator for detection [8]. In our study, immunofluorescence showed a higher level of CA‐IX, a marker for hypoxia, and correspondingly a lower level of PD‐L1 in the hypoxia^high^/immune^low^ group.

Interestingly, both the activating and inhibitory immune markers were highly expressed in the hypoxia^low^/immune^high^ group, which seems contradictory in common sense. This result may indicate a consuming anticancer immune response in the hypoxia^low^/immune^high^ group with an increased anti‐immune reaction in tumor cells. The active anticancer immune response may be restored through immune checkpoint blockade. As described above, myeloid‐derived suppressor cells (MDSCs) and TAMs may be regulated by the hypoxia status and contribute to immunosuppression.

Overall, hypoxia exerts its function on tumor cells and the TME, tipping the balance of the immune response of chemokines, immune effector production, and immune cell infiltration. For patients with a relatively hypoxia‐high/immune‐low feature, hypoxic modifier combined with subsequent immunotherapies is potentially applicable.

There are certain limitations to our study. First, this is a cross‐cohort and retrospective study, and further prospective analysis in multicenter cohorts is necessary due to the observed heterogeneity between different populations. Second, although a prognostic continuous index has been developed, further inclusion of clinical factors (TNM stage, histological grade, age at diagnosis, and anatomic site) may minimize the potential of bias. Third, this is a retrospective study, and the size of the SYSUCC cohort was relatively small; only stromal PD‐L1 was analyzed. Finally, qRT‐PCR analysis of all selected signatures might be costly in clinical practice, and more convenient methods could be further applied, such as IHC using CA‐IX and relevant immune markers.

The development of a novel comprehensive hypoxia–immune status classifier in our study implies that individual treatment should be applied in different subgroups. For example, immunotherapies might be more favorable in patients with hypoxia^low^/immune^high^ features. A higher CIHI was indicative of an active immune response and potential benefit from immune checkpoint blockade, such as anti‐PD1/PD‐L1 treatment. For patients in the hypoxia^high^/immune^low^ group with a poorer prognosis, a lower CIHI might imply a hypoxic microenvironment and lower efficacy for immunotherapy. However, hypoxia modifiers might help to sensitize patients to further radiotherapy and chemotherapy after surgery. The results demonstrated that subsequent immunotherapy might work after hypoxia modification. Further subsequent immunotherapy might prevent the immunosuppressive response after treatment. For those with an intermediate CIHI exhibiting a mixed status of hypoxia and immune response, both anti‐hypoxia and immunomodulatory drugs may harbor certain effects on patients.

## 5 Conclusions

In conclusion, a novel prognostic index and classifier based on hypoxia and immune expression profiles were developed and cross‐cohort validated. This classifier could be used for prognostic prediction and selecting patients for hypoxia modification and immunotherapies, such as PD‐L1/PD‐1 and CTLA‐4 blockade. We recommend further validating the efficacy of the CIHI in prospective studies and developing a simplified version using more convenient methods, such as IHC.

## Authors contributions

XX and ZL conceived and designed the study. SZ, YZ, JL, and WX developed the methodology. SZ, JL, TM, and SL involved in data acquisition. JL, SZ, and YZ performed bioinformatic analysis. SZ, YZ, and JL analyzed and interpreted the data. SZ, YZ, JL, and AY performed the experiments. XX and ZL supervised the study. All authors read and approved the final manuscript.

## Conflict of interest

The authors declare no conflict of interest.

## Supporting information


**Fig S1.** Detailed flowchart of this study.

**Fig S2.** Kaplan–Meier analysis of the genes included for CIHI construction (TAPBPL, CXCL11, HLA‐A, TCF7L2, TANK, IL12B, IL18RAP, PFKL, SLC25A1, and SERPINE1).

**Fig S3.** (a, b) Comparison of 22 infiltrating immune cells in different subgroups of the TCGA and GSE58812 cohorts.

**Fig S4.** Decision curve analysis of the CIHI in the METABRIC, TCGA, and GSE58812 cohorts.

**Fig S5.** (a, b) Validation of the prognostic value of the CIHI for OS and DFS in the SYSUCC cohort.

**Fig S6.** (a, b) Univariate and multivariate Cox regression analyses of the CIHI and clinical indicators.

**Fig S7.** (a, b) Correlation between the CIHI and tumor size in the METABRIC cohort or AJCC T stage in the TCGA cohort.

**Fig S8.** (a) Box and whisker plots showing the expression of the selected hypoxia‐related genes in the FUSCCTNBC cohort.

**Fig S9.** Correlation between the CIHI and previously reported hypoxia scores in the TCGA‐TNBC cohort.

**Table S1.** Primers used in this study.

**Table S2.** Reagents used in this study.

**Table S3.** Correlation between CIHI and xCell results.

**Appendix S1.** Cell cycle correlation.

**Appendix S2.** Cell proliferation correlation.

## References

[1] DeSantis CE , Ma J , Gaudet MM , Newman LA , Miller KD , Goding Sauer A , Jemal A and Siegel RL (2019) Breast cancer statistics, 2019. CA Cancer J Clin 69, 438–451. 31577379 10.3322/caac.21583

[2] Siegel RL , Miller KD and Jemal A . (2020) Cancer statistics, 2020. CA J Clin 70, 7–30. 10.3322/caac.21590 31912902

[3] Dent R , Trudeau M , Pritchard KI , Hanna WM , Kahn HK , Sawka CA , Lickley LA , Rawlinson E , Sun P and Narod SA (2007) Triple‐negative breast cancer: clinical features and patterns of recurrence. Clin Cancer Res 13, 4429–4434. 17671126 10.1158/1078-0432.CCR-06-3045

[4] Waks AG and Winer EP (2019) Breast cancer treatment: a review. JAMA 321, 288–300. 30667505 10.1001/jama.2018.19323

[5] Lehmann BD , Bauer JA , Chen X , Sanders ME , Chakravarthy AB , Shyr Y and Pietenpol JA (2011) Identification of human triple‐negative breast cancer subtypes and preclinical models for selection of targeted therapies. J Clin Invest 121, 2750–2767. 21633166 10.1172/JCI45014 PMC3127435

[6] Bareche Y , Venet D , Ignatiadis M , Aftimos P , Piccart M , Rothe F and Sotiriou C (2018) Unravelling triple‐negative breast cancer molecular heterogeneity using an integrative multiomic analysis. Ann Oncol 29, 895–902. 29365031 10.1093/annonc/mdy024 PMC5913636

[7] Adams S , Diamond JR , Hamilton E , Pohlmann PR , Tolaney SM , Chang CW , Zhang W , Iizuka K , Foster PG , Molinero L *et al* (2018) Atezolizumab plus nab‐paclitaxel in the treatment of metastatic triple‐negative breast cancer with 2‐year survival follow‐up. JAMA Oncol 5, 334 10.1001/jamaoncol.2018.5152 PMC6439843 30347025

[8] Schmid P , Adams S , Rugo HS , Schneeweiss A , Barrios CH , Iwata H , Dieras V , Hegg R , Im SA , Shaw Wright G *et al* (2018) Atezolizumab and Nab‐Paclitaxel in advanced triple‐negative breast cancer. N Engl J Med 379, 2108–2121. 30345906 10.1056/NEJMoa1809615

[9] Loibl S , Untch M , Burchardi N , Huober J , Sinn BV , Blohmer JU , Grischke EM , Furlanetto J , Tesch H , Hanusch C *et al* (2019) A randomised phase II study investigating durvalumab in addition to an anthracycline taxane‐based neoadjuvant therapy in early triple negative breast cancer – clinical results and biomarker analysis of GeparNuevo study. Ann Oncol 30, 1279–1288. 31095287 10.1093/annonc/mdz158

[10] Miller LD , Chou JA , Black MA , Print C , Chifman J , Alistar A , Putti T , Zhou X , Bedognetti D , Hendrickx W *et al* (2016) Immunogenic subtypes of breast cancer delineated by gene classifiers of immune responsiveness. Cancer Immunol Res 4, 600–610. 27197066 10.1158/2326-6066.CIR-15-0149 PMC4930674

[11] Yang S , Wu Y , Deng Y , Zhou L , Yang P , Zheng Y , Zhang D , Zhai Z , Li N , Hao Q *et al* (2019) Identification of a prognostic immune signature for cervical cancer to predict survival and response to immune checkpoint inhibitors. Oncoimmunology 8, e1659094. 31741756 10.1080/2162402X.2019.1659094 PMC6844304

[12] Gilkes DM , Semenza GL and Wirtz D (2014) Hypoxia and the extracellular matrix: drivers of tumour metastasis. Nat Rev Cancer 14, 430–439. 24827502 10.1038/nrc3726 PMC4283800

[13] Rankin EB and Giaccia AJ (2016) Hypoxic control of metastasis. Science 352, 175–180. 27124451 10.1126/science.aaf4405 PMC4898055

[14] Simon F , Bockhorn M , Praha C , Baba HA , Broelsch CE , Frilling A and Weber F (2010) Deregulation of HIF1‐alpha and hypoxia‐regulated pathways in hepatocellular carcinoma and corresponding non‐malignant liver tissue–influence of a modulated host stroma on the prognosis of HCC. Langenbecks Arch Surg 395, 395–405. 20165955 10.1007/s00423-009-0590-9

[15] Mathieu J , Zhang Z , Zhou W , Wang AJ , Heddleston JM , Pinna CM , Hubaud A , Stadler B , Choi M , Bar M *et al* (2011) HIF induces human embryonic stem cell markers in cancer cells. Cancer Res 71, 4640–4652. 21712410 10.1158/0008-5472.CAN-10-3320 PMC3129496

[16] Semenza GL (2010) HIF‐1: upstream and downstream of cancer metabolism. Curr Opin Genet Dev 20, 51–56. 19942427 10.1016/j.gde.2009.10.009 PMC2822127

[17] Semenza GL (2017) Hypoxia‐inducible factors: coupling glucose metabolism and redox regulation with induction of the breast cancer stem cell phenotype. The EMBO J 36, 252–259. 28007895 10.15252/embj.201695204 PMC5286373

[18] Batie M , Frost J , Frost M , Wilson JW , Schofield P and Rocha S (2019) Hypoxia induces rapid changes to histone methylation and reprograms chromatin. Science (New York, N.Y.) 363, 1222–1226. 10.1126/science.aau5870 30872526

[19] Godet I , Shin YJ , Ju JA , Ye IC , Wang G and Gilkes DM (2019) Fate‐mapping post‐hypoxic tumor cells reveals a ROS‐resistant phenotype that promotes metastasis. Nat Commun 10, 4862. 31649238 10.1038/s41467-019-12412-1 PMC6813355

[20] Li H , Rokavec M , Jiang L , Horst D and Hermeking H (2017) Antagonistic effects of p53 and HIF1A on microRNA‐34a regulation of PPP1R11 and STAT3 and hypoxia‐induced epithelial to mesenchymal transition in colorectal cancer cells. Gastroenterology 153, 505–520. 28435028 10.1053/j.gastro.2017.04.017

[21] Palazón A , Martínez‐Forero I , Teijeira A , Morales‐Kastresana A , Alfaro C , Sanmamed MF , Perez‐Gracia JL , Peñuelas I , Hervás‐Stubbs S , Rouzaut A *et al* (2012) The HIF‐1α hypoxia response in tumor‐infiltrating T lymphocytes induces functional CD137 (4–1BB) for immunotherapy. Cancer Discov 2, 608–623. 22719018 10.1158/2159-8290.CD-11-0314

[22] Chen F , Chen J , Yang L , Liu J , Zhang X , Zhang Y , Tu Q , Yin D , Lin D , Wong PP *et al* (2019) Extracellular vesicle‐packaged HIF‐1alpha‐stabilizing lncRNA from tumour‐associated macrophages regulates aerobic glycolysis of breast cancer cells. Nat Cell Biol 21, 498–510. 30936474 10.1038/s41556-019-0299-0

[23] Ma B , Cheng H , Mu C , Geng G , Zhao T , Luo Q , Ma K , Chang R , Liu Q , Gao R *et al* (2019) The SIAH2‐NRF1 axis spatially regulates tumor microenvironment remodeling for tumor progression. Nat Commun 10, 1034. 30833558 10.1038/s41467-019-08618-y PMC6399320

[24] Graham C , Barsoum I , Kim J , Black M and Siemens RD (2015) Mechanisms of hypoxia‐induced immune escape in cancer and their regulation by nitric oxide. Redox Biol 5, 417. 10.1016/j.redox.2015.09.022 28162279

[25] Curtis C , Shah SP , Chin S‐F , Turashvili G , Rueda OM , Dunning MJ , Speed D , Lynch AG , Samarajiwa S , Yuan Y , *et al* (2012) The genomic and transcriptomic architecture of 2,000 breast tumours reveals novel subgroups. Nature 486, 346–352. 22522925 10.1038/nature10983 PMC3440846

[26] Xiao Y , Ma D , Zhao S , Suo C , Shi J , Xue MZ , Ruan M , Wang H , Zhao J , Li Q *et al* (2019) Multi‐omics profiling reveals distinct microenvironment characterization and suggests immune escape mechanisms of triple‐negative breast cancer. Clin Cancer Res 25, 5002–5014. 30837276 10.1158/1078-0432.CCR-18-3524

[27] Ritchie ME , Phipson B , Wu D , Hu Y , Law CW , Shi W and Smyth GK (2015) limma powers differential expression analyses for RNA‐sequencing and microarray studies. Nucleic Acids Res 43, e47. 25605792 10.1093/nar/gkv007 PMC4402510

[28] Jezequel P , Loussouarn D , Guerin‐Charbonnel C , Campion L , Vanier A , Gouraud W , Lasla H , Guette C , Valo I , Verriele V and *et al* (2015) Gene‐expression molecular subtyping of triple‐negative breast cancer tumours: importance of immune response. Breast Cancer Res 17, 43. 25887482 10.1186/s13058-015-0550-y PMC4389408

[29] Gu Z , Gu L , Eils R , Schlesner M and Brors B (2014) circlize Implements and enhances circular visualization in R. Bioinformatics (Oxford, England) 30, 2811–2812. 10.1093/bioinformatics/btu393 24930139

[30] Bhandari V , Hoey C , Liu LY , Lalonde E , Ray J , Livingstone J , Lesurf R , Shiah YJ , Vujcic T , Huang X *et al* (2019) Molecular landmarks of tumor hypoxia across cancer types. Nat Genet 51, 308–318. 30643250 10.1038/s41588-018-0318-2

[31] Buffa FM , Harris AL , West CM and Miller CJ (2010) Large meta‐analysis of multiple cancers reveals a common, compact and highly prognostic hypoxia metagene. Br J Cancer 102, 428–435. 20087356 10.1038/sj.bjc.6605450 PMC2816644

[32] Eustace A , Mani N , Span PN , Irlam JJ , Taylor J , Betts GN , Denley H , Miller CJ , Homer JJ , Rojas AM *et al* (2013) A 26‐gene hypoxia signature predicts benefit from hypoxia‐modifying therapy in laryngeal cancer but not bladder cancer. Clin Cancer Res 19, 4879–4888. 23820108 10.1158/1078-0432.CCR-13-0542 PMC3797516

[33] Hu Z , Fan C , Livasy C , He X , Oh DS , Ewend MG , Carey LA , Subramanian S , West R , Ikpatt F *et al* (2009) A compact VEGF signature associated with distant metastases and poor outcomes. BMC Med 7, 9. 19291283 10.1186/1741-7015-7-9 PMC2671523

[34] Winter SC , Buffa FM , Silva P , Miller C , Valentine HR , Turley H , Shah KA , Cox GJ , Corbridge RJ , Homer JJ *et al* (2007) Relation of a hypoxia metagene derived from head and neck cancer to prognosis of multiple cancers. Cancer Res 67, 3441–3449. 17409455 10.1158/0008-5472.CAN-06-3322

[35] Tibshirani R (1997) The lasso method for variable selection in the Cox model. Stat Med 16, 385–395. 9044528 10.1002/(sici)1097-0258(19970228)16:4<385::aid-sim380>3.0.co;2-3

[36] Friedman J , Hastie T and Tibshirani R (2010) Regularization paths for generalized linear models via coordinate descent. J Stat Softw 33, 1–22. 20808728 PMC2929880

[37] Newman AM , Liu CL , Green MR , Gentles AJ , Feng W , Xu Y , Hoang CD , Diehn M and Alizadeh AA (2015) Robust enumeration of cell subsets from tissue expression profiles. Nat Methods 12, 453–457. 25822800 10.1038/nmeth.3337 PMC4739640

[38] Aran D , Hu Z and Butte AJ (2017) xCell: digitally portraying the tissue cellular heterogeneity landscape. Genome Biol 18, 220. 29141660 10.1186/s13059-017-1349-1 PMC5688663

[39] Yoshihara K , Shahmoradgoli M , Martinez E , Vegesna R , Kim H , Torres‐Garcia W , Trevino V , Shen H , Laird PW , Levine DA *et al* (2013) Inferring tumour purity and stromal and immune cell admixture from expression data. Nat Commun 4, 2612. 24113773 10.1038/ncomms3612 PMC3826632

[40] Becht E , Giraldo NA , Lacroix L , Buttard B , Elarouci N , Petitprez F , Selves J , Laurent‐Puig P , Sautes‐Fridman C , Fridman WH *et al* (2016) Estimating the population abundance of tissue‐infiltrating immune and stromal cell populations using gene expression. Genome Biol 17, 218. 27765066 10.1186/s13059-016-1070-5 PMC5073889

[41] Hänzelmann S , Castelo R and Guinney J (2013) GSVA: gene set variation analysis for microarray and RNA‐seq data. BMC Bioinformatics 14, 7. 23323831 10.1186/1471-2105-14-7 PMC3618321

[42] Mayakonda A , Lin DC , Assenov Y , Plass C and Koeffler HP (2018) Maftools: efficient and comprehensive analysis of somatic variants in cancer. Genome Res 28, 1747–1756. 30341162 10.1101/gr.239244.118 PMC6211645

[43] Xiao W , Zheng S , Zou Y , Yang A , Xie X , Tang H and Xie X (2019) CircAHNAK1 inhibits proliferation and metastasis of triple‐negative breast cancer by modulating miR‐421 and RASA1. Aging (Albany NY) 11, 12043–12056. 31857500 10.18632/aging.102539 PMC6949091

[44] Blanche P , Dartigues JF and Jacqmin‐Gadda H (2013) Estimating and comparing time‐dependent areas under receiver operating characteristic curves for censored event times with competing risks. Stat Med 32, 5381–5397. 24027076 10.1002/sim.5958

[45] Vickers AJ , Cronin AM , Elkin EB and Gonen M (2008) Extensions to decision curve analysis, a novel method for evaluating diagnostic tests, prediction models and molecular markers. BMC Med Inform Decis Mak 8, 53. 19036144 10.1186/1472-6947-8-53 PMC2611975

[46] Wu M‐Z , Cheng W‐C , Chen S‐F , Nieh S , O'Connor C , Liu C‐L , Tsai W‐W , Wu C‐J , Martin L , Lin Y‐S *et al* (2017) miR‐25/93 mediates hypoxia‐induced immunosuppression by repressing cGAS. Nat Cell Biol 19, 1286–1296. 28920955 10.1038/ncb3615 PMC5658024

[47] Wu Q , Zhou W , Yin S , Zhou Y , Chen T , Qian J , Su R , Hong L , Lu H , Zhang F *et al* (2019) Blocking triggering receptor expressed on myeloid cells‐1‐positive tumor‐associated macrophages induced by hypoxia reverses immunosuppression and anti‐programmed cell death ligand 1 resistance in liver cancer. Hepatology 70, 198–214. 30810243 10.1002/hep.30593 PMC6618281

[48] Brooks JM , Menezes AN , Ibrahim M , Archer L , Lal N , Bagnall CJ , von Zeidler SV , Valentine HR , Spruce RJ , Batis N *et al* (2019) Development and validation of a combined hypoxia and immune prognostic classifier for head and neck cancer. Clin Cancer Res 25, 5315–5328. 31182433 10.1158/1078-0432.CCR-18-3314

[49] Bartosova M , Parkkila S , Pohlodek K , Karttunen TJ , Galbavy S , Mucha V , Harris AL , Pastorek J and Pastorekova S (2002) Expression of carbonic anhydrase IX in breast is associated with malignant tissues and is related to overexpression of c‐erbB2. J Pathol 197, 314–321. 12115877 10.1002/path.1120

[50] Eom KY , Jang MH , Park SY , Kang EY , Kim SW , Kim JH , Kim JS and Kim IA (2016) The expression of carbonic anhydrase (CA) IX/XII and lymph node metastasis in early breast cancer. Cancer Res Treat 48, 125–132. 25761481 10.4143/crt.2014.243 PMC4720081

[51] Tafreshi NK , Lloyd MC , Proemsey JB , Bui MM , Kim J , Gillies RJ and Morse DL (2016) Evaluation of CAIX and CAXII expression in breast cancer at varied O_2_ levels: CAIX is the superior surrogate imaging biomarker of tumor hypoxia. Mol Imaging Biol 18, 219–231. 26276155 10.1007/s11307-015-0885-x PMC4754166

[52] Baxevanis CN , Fortis SP and Perez SA . (2019) The balance between breast cancer and the immune system: Challenges for prognosis and clinical benefit from immunotherapies. Sem Cancer Biol. 10.1016/j.semcancer.2019.12.018 31881337

[53] Shao C , Yang F , Miao S , Liu W , Wang C , Shu Y and Shen H (2018) Role of hypoxia‐induced exosomes in tumor biology. Mol Cancer 17, 120. 30098600 10.1186/s12943-018-0869-y PMC6087002

[54] Palazon A , Tyrakis PA , Macias D , Veliça P , Rundqvist H , Fitzpatrick S , Vojnovic N , Phan AT , Loman N , Hedenfalk I , *et al* (2017) An HIF‐1α/VEGF‐A axis in cytotoxic T cells regulates tumor progression. Cancer Cell 32, 669–683.e5. 29136509 10.1016/j.ccell.2017.10.003 PMC5691891

[55] Chang C‐H , Qiu J , O'Sullivan D , Buck MD , Noguchi T , Curtis JD , Chen Q , Gindin M , Gubin MM , van der Windt GJW *et al* (2015) Metabolic competition in the tumor microenvironment is a driver of cancer progression. Cell 162, 1229–1241. 26321679 10.1016/j.cell.2015.08.016 PMC4864363

[56] Ho P‐C , Bihuniak JD , Macintyre AN , Staron M , Liu X , Amezquita R , Tsui Y‐C , Cui G , Micevic G , Perales JC *et al* (2015) Phosphoenolpyruvate is a metabolic checkpoint of anti‐tumor T Cell responses. Cell 162, 1217–1228. 26321681 10.1016/j.cell.2015.08.012 PMC4567953

[57] Amini MA , Abbasi AZ , Cai P , Lip H , Gordijo CR , Li J , Chen B , Zhang L , Rauth AM and Wu XY (2019) Combining tumor microenvironment modulating nanoparticles with doxorubicin to enhance chemotherapeutic efficacy and boost antitumor immunity. J Natl Cancer Inst 111, 399–408. 30239773 10.1093/jnci/djy131

[58] Schmid P , Rugo HS , Adams S , Schneeweiss A , Barrios CH , Iwata H , Diéras V , Henschel V , Molinero L , Chui SY *et al* (2020) Atezolizumab plus nab‐paclitaxel as first‐line treatment for unresectable, locally advanced or metastatic triple‐negative breast cancer (IMpassion130): updated efficacy results from a randomised, double‐blind, placebo‐controlled, phase 3 trial. Lancet Oncol 21, 44–59. 31786121 10.1016/S1470-2045(19)30689-8

[59] DeNardo DG and Ruffell B (2019) Macrophages as regulators of tumour immunity and immunotherapy. Nat Rev Immunol 19, 369–382. 30718830 10.1038/s41577-019-0127-6 PMC7339861

[60] Neo SY , Yang Y , Record J , Ma R , Chen X , Chen Z , Tobin NP , Blake E , Seitz C , Thomas R *et al* (2020) CD73 immune checkpoint defines regulatory NK cells within the tumor microenvironment. J Clin Invest 130, 1185–1198. 31770109 10.1172/JCI128895 PMC7269592

[61] Speiser DE , Ho P‐C and Verdeil G (2016) Regulatory circuits of T cell function in cancer. Nat Rev Immunol 16, 599–611. 27526640 10.1038/nri.2016.80

[62] Matikas A , Zerdes I , Lovrot J , Richard F , Sotiriou C , Bergh J , Valachis A and Foukakis T (2019) Prognostic implications of PD‐L1 expression in breast cancer: systematic review and meta‐analysis of immunohistochemistry and pooled analysis of transcriptomic data. Clin Cancer Res 25, 5717–5726. 31227501 10.1158/1078-0432.CCR-19-1131

[63] Sobral‐Leite M , Van de Vijver K , Michaut M , van der Linden R , Hooijer GKJ , Horlings HM , Severson TM , Mulligan AM , Weerasooriya N , Sanders J *et al* (2018) Assessment of PD‐L1 expression across breast cancer molecular subtypes, in relation to mutation rate, BRCA1‐like status, tumor‐infiltrating immune cells and survival. Oncoimmunology 7, e1509820. 30524905 10.1080/2162402X.2018.1509820 PMC6279322

[64] Cimino‐Mathews A , Thompson E , Taube JM , Ye X , Lu Y , Meeker A , Xu H , Sharma R , Lecksell K , Cornish TC *et al* (2016) PD‐L1 (B7–H1) expression and the immune tumor microenvironment in primary and metastatic breast carcinomas. Hum Pathol 47, 52–63. 26527522 10.1016/j.humpath.2015.09.003 PMC4778421

