# Supplementary figures and images for: Identification and validation of a combined hypoxia and immune index for triple‐negative breast cancer
Source: Mol Oncol. 2020 Jul 1;14(11):2814–33. doi: 10.1002/1878-0261.12747 (PMC7607163; doi:10.1002/1878-0261.12747)

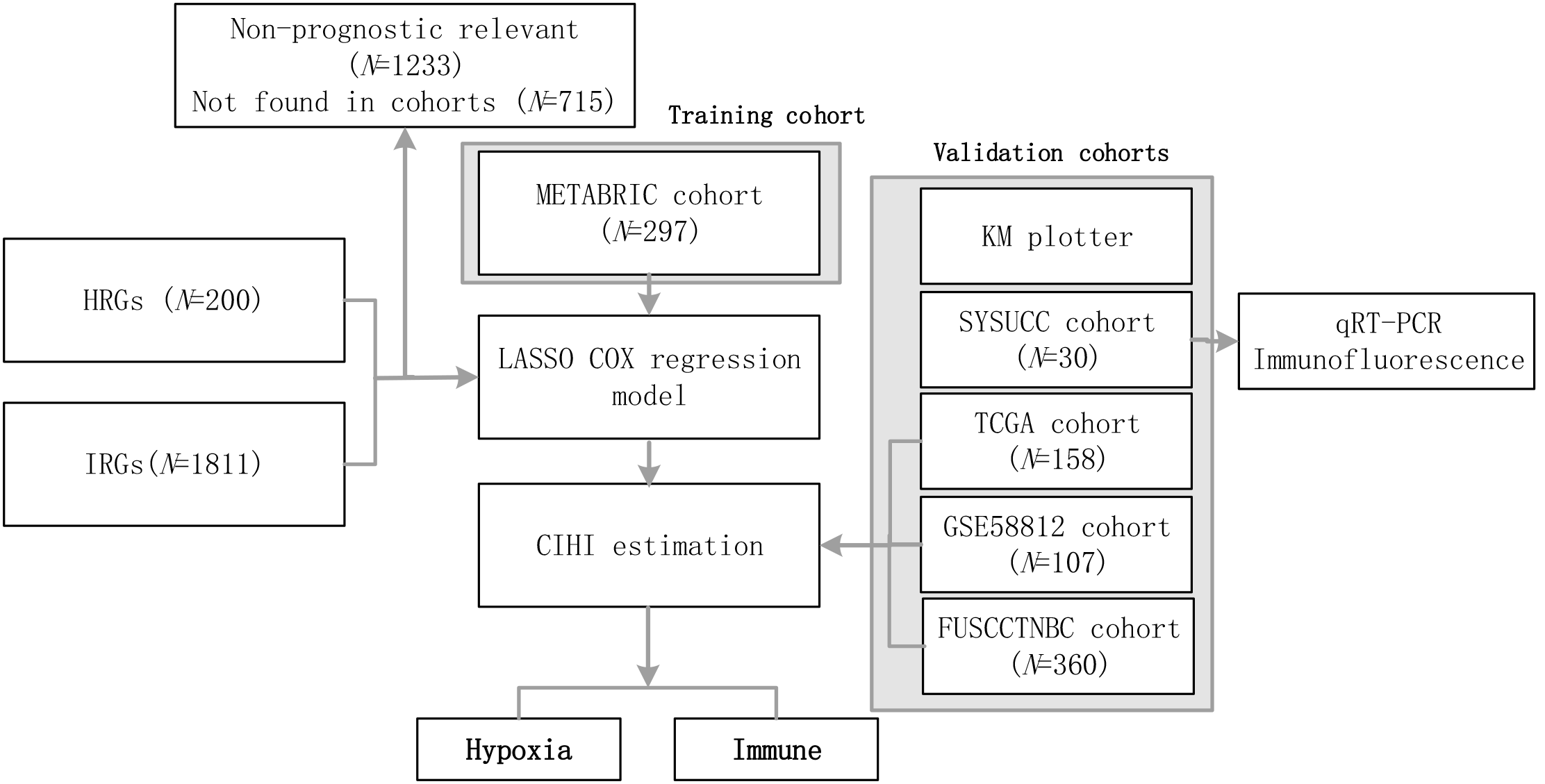

Supplement: Supplementary file 1 — Fig S1. Detailed flowchart of this study. [file MOL2-14-2814-s001.tif]

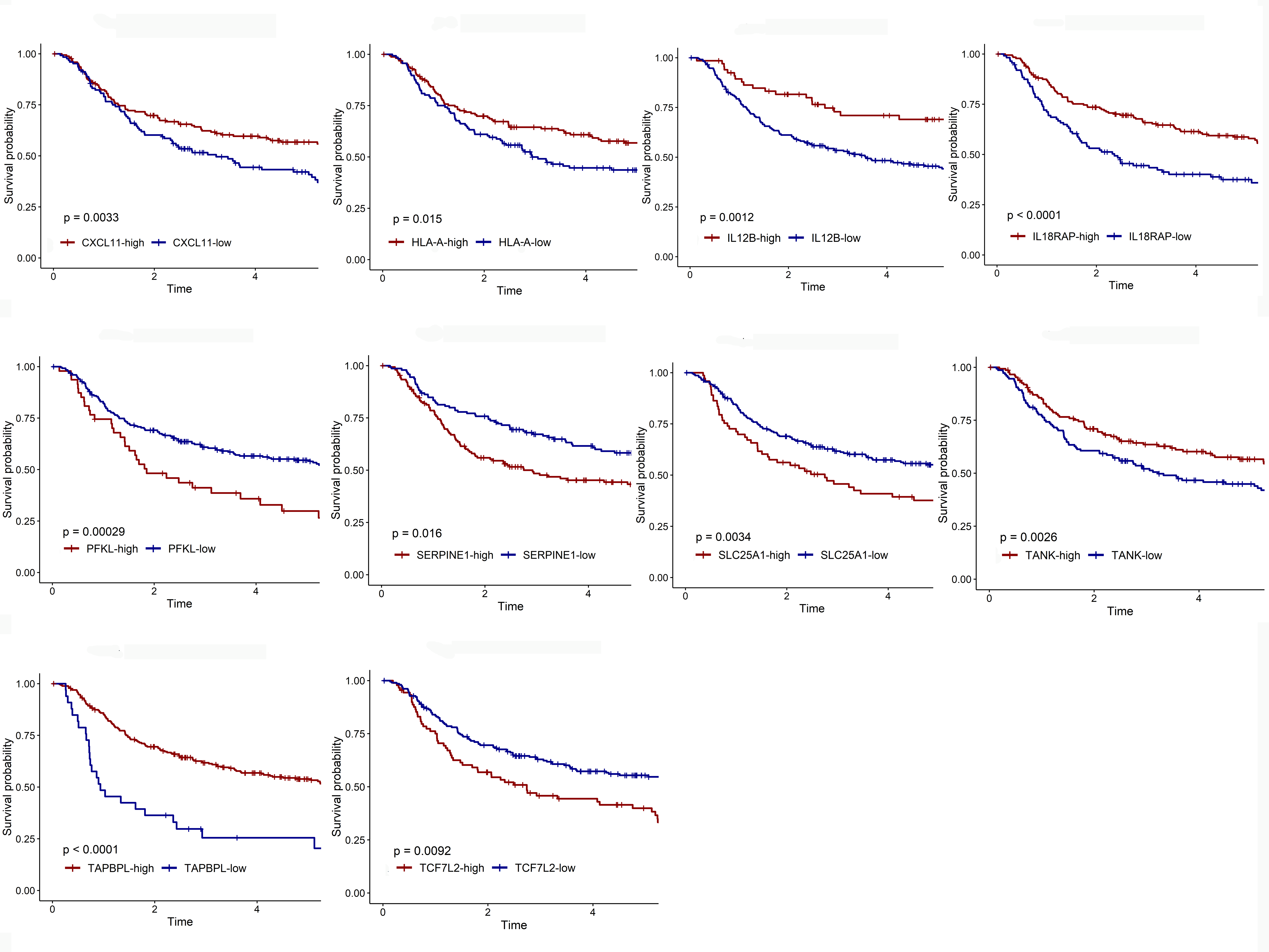

Supplement: Supplementary file 2 — Fig S2. Kaplan–Meier analysis of the genes included for CIHI construction (TAPBPL, CXCL11, HLA‐A, TCF7L2, TANK, IL12B, IL18RAP, PFKL, SLC25A1, and SERPINE1). [file MOL2-14-2814-s002.tif]

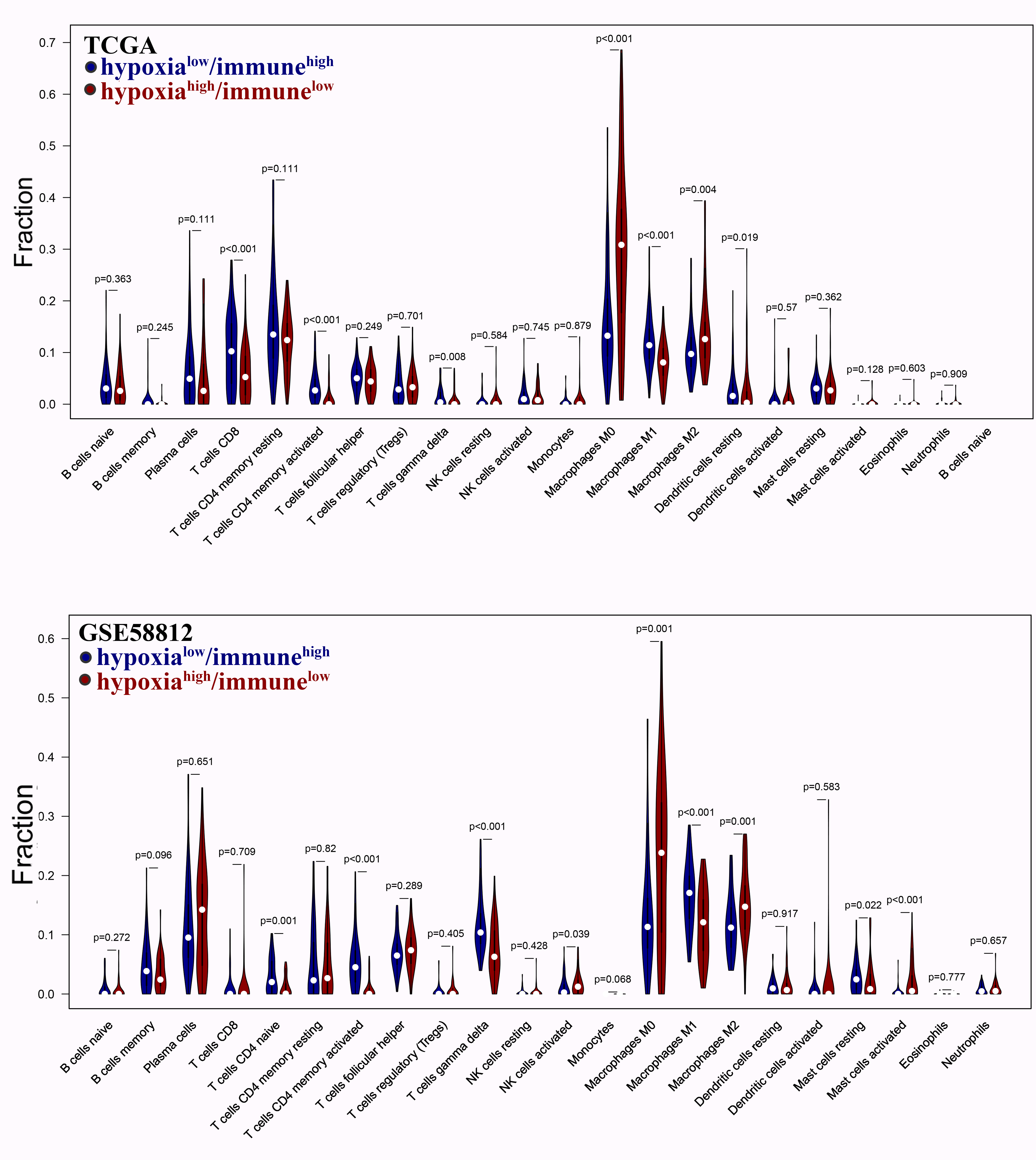

Supplement: Supplementary file 3 — Fig S3. (a, b) Comparison of 22 infiltrating immune cells in different subgroups of the TCGA and GSE58812 cohorts. [file MOL2-14-2814-s003.tif]

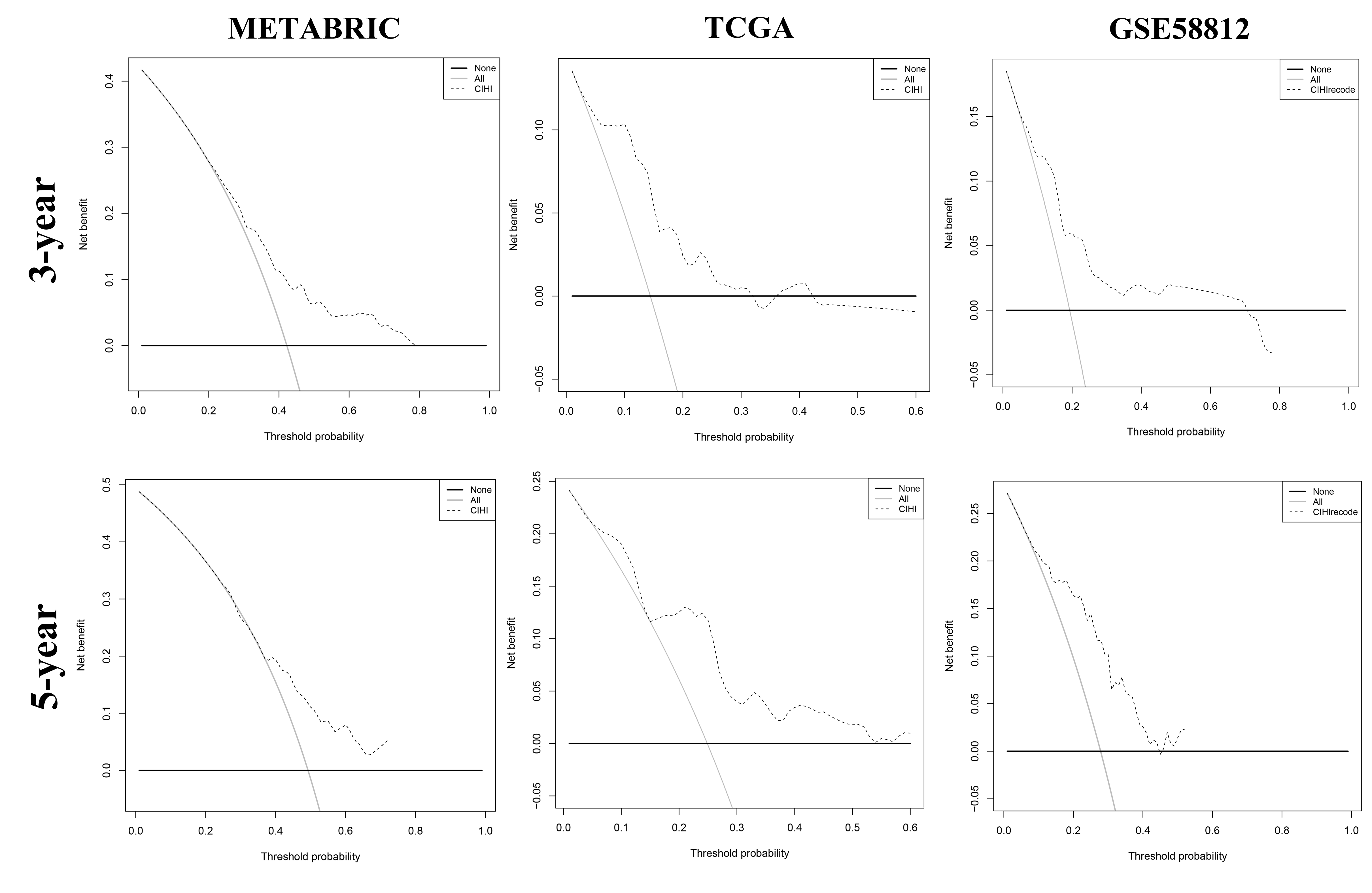

Supplement: Supplementary file 4 — Fig S4. Decision curve analysis of the CIHI in the METABRIC, TCGA, and GSE58812 cohorts. [file MOL2-14-2814-s004.tif]

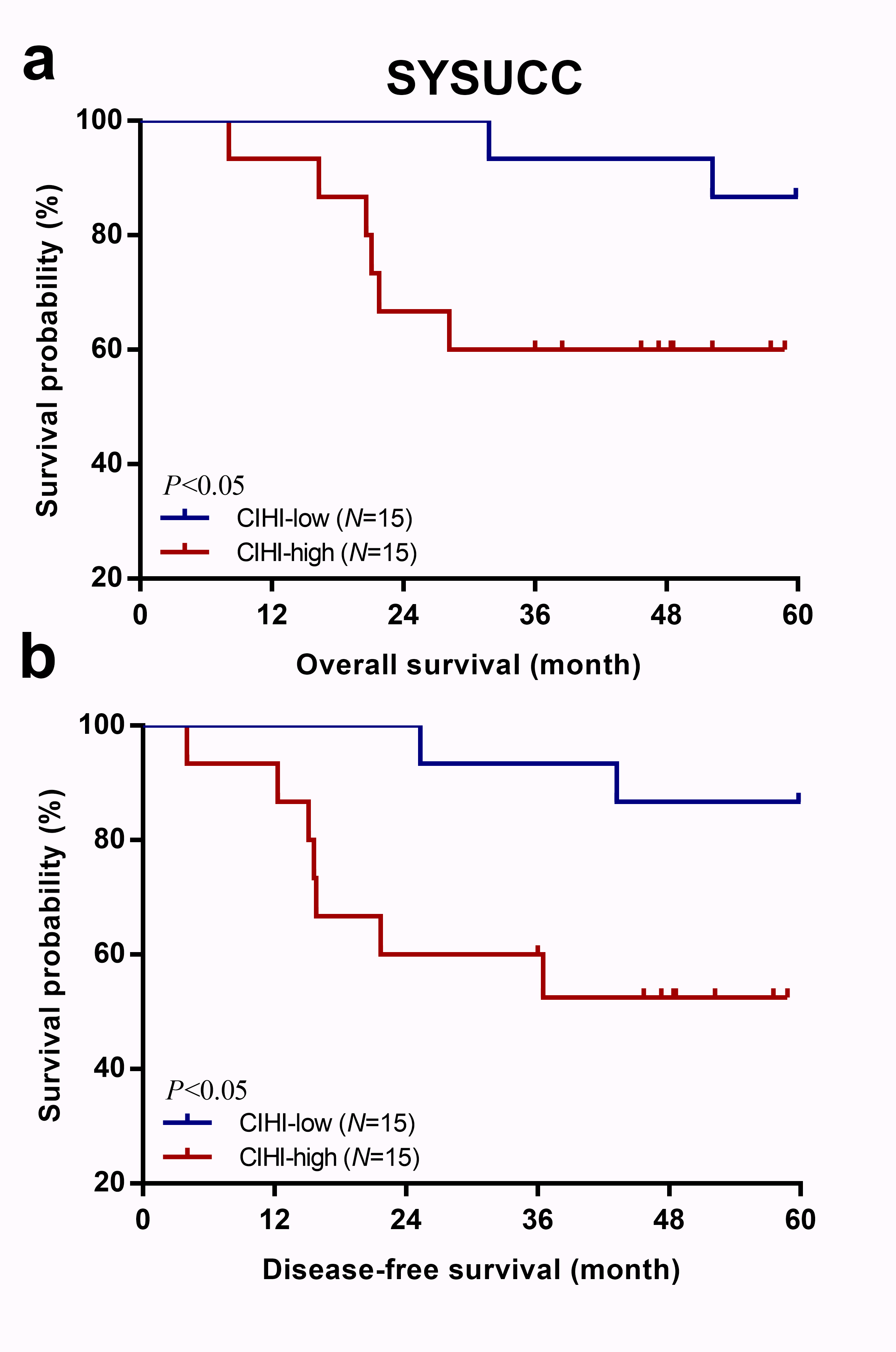

Supplement: Supplementary file 5 — Fig S5. (a, b) Validation of the prognostic value of the CIHI for OS and DFS in the SYSUCC cohort. [file MOL2-14-2814-s005.tif]

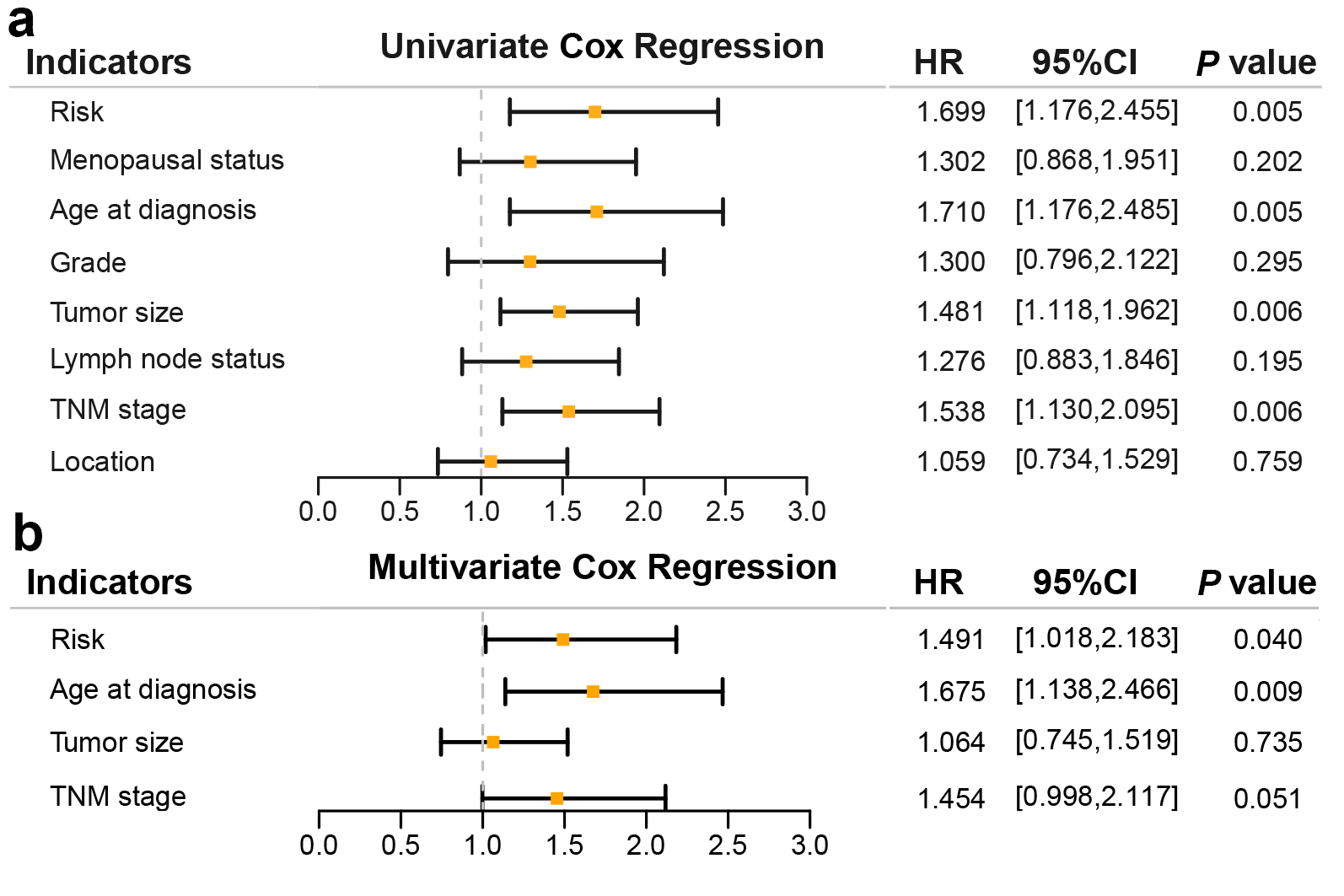

Supplement: Supplementary file 6 — Fig S6. (a, b) Univariate and multivariate Cox regression analyses of the CIHI and clinical indicators. [file MOL2-14-2814-s006.tif]

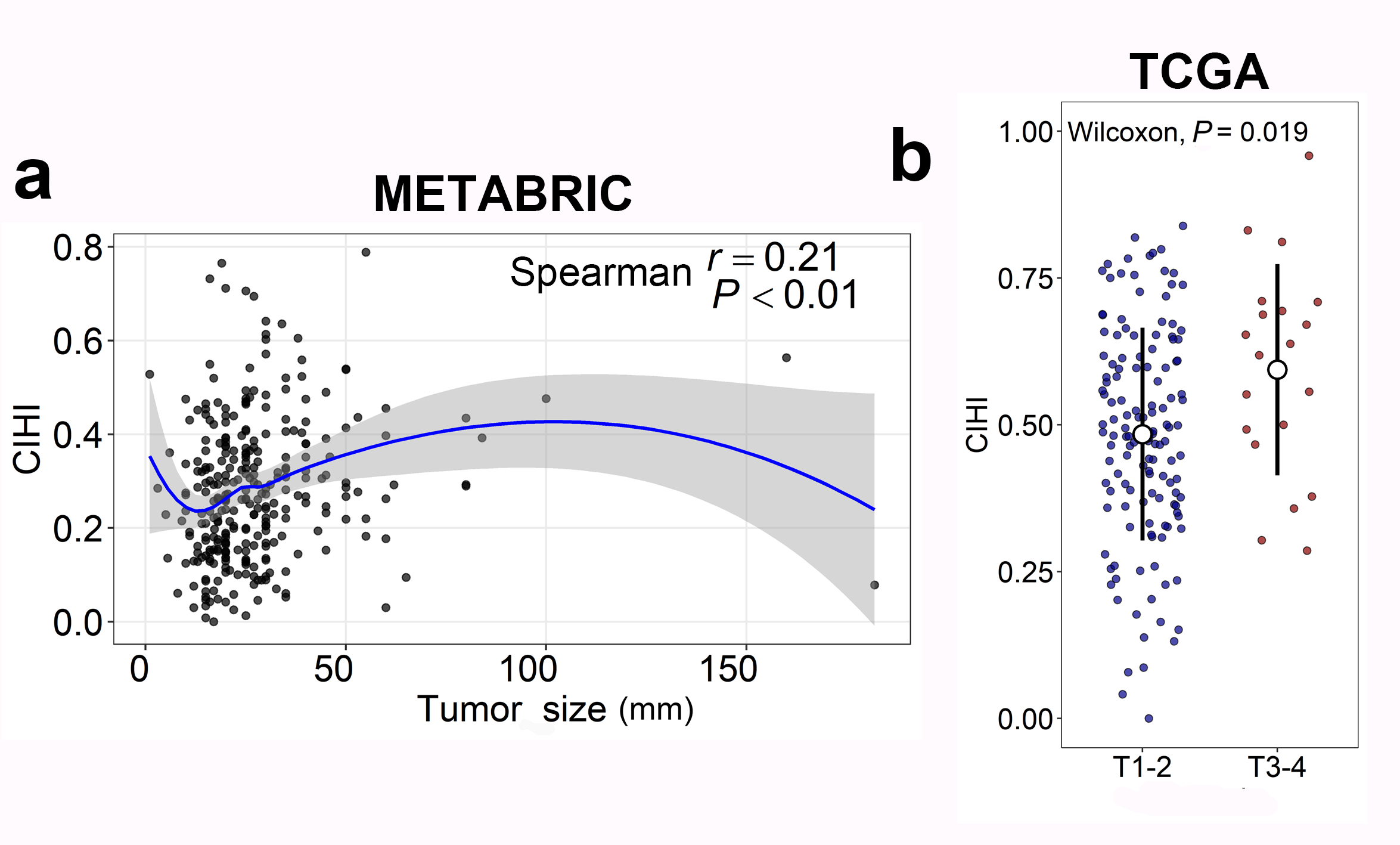

Supplement: Supplementary file 7 — Fig S7. (a, b) Correlation between the CIHI and tumor size in the METABRIC cohort or AJCC T stage in the TCGA cohort. [file MOL2-14-2814-s007.tif]

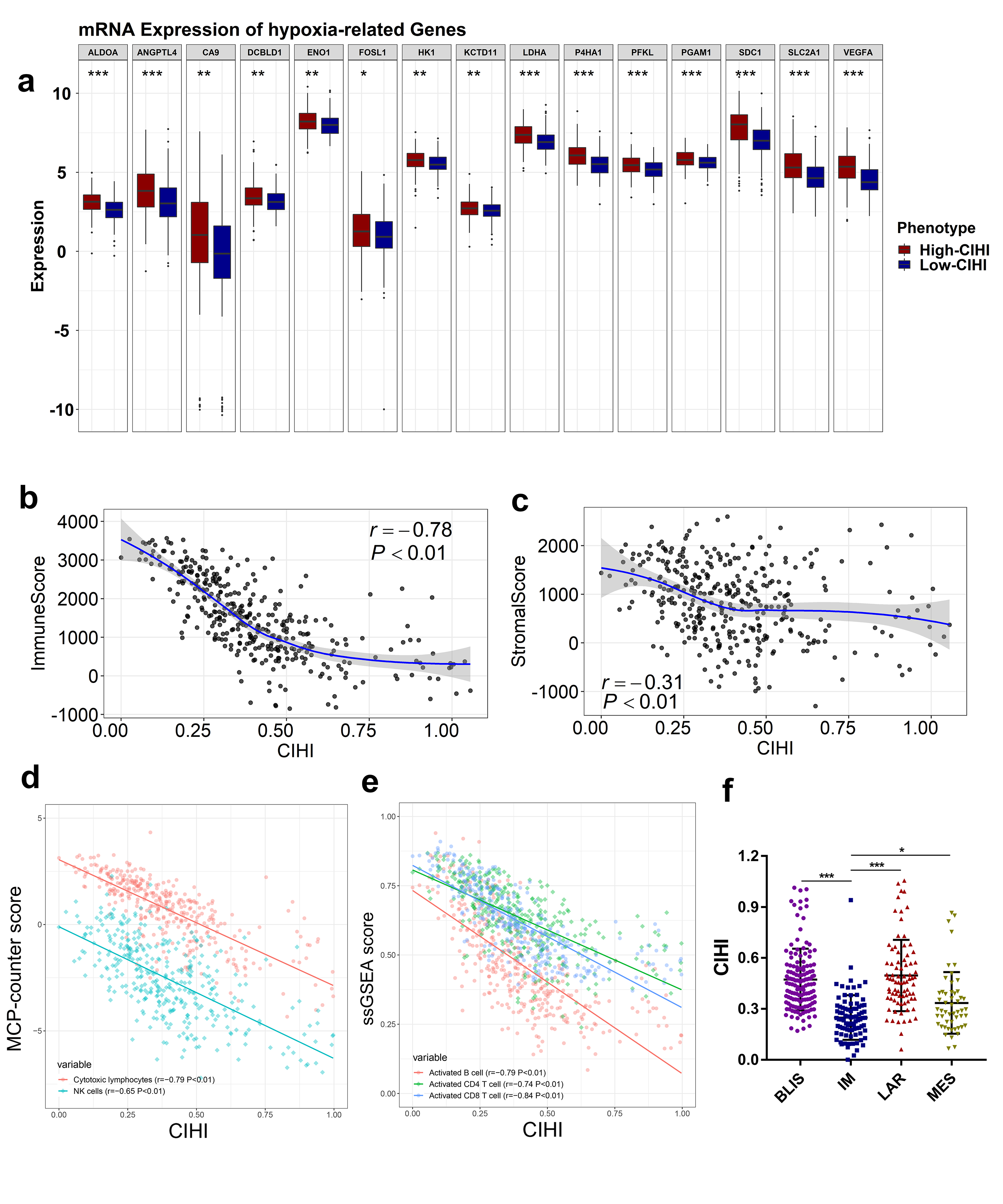

Supplement: Supplementary file 8 — Fig S8. (a) Box and whisker plots showing the expression of the selected hypoxia‐related genes in the FUSCCTNBC cohort. [file MOL2-14-2814-s008.tif]

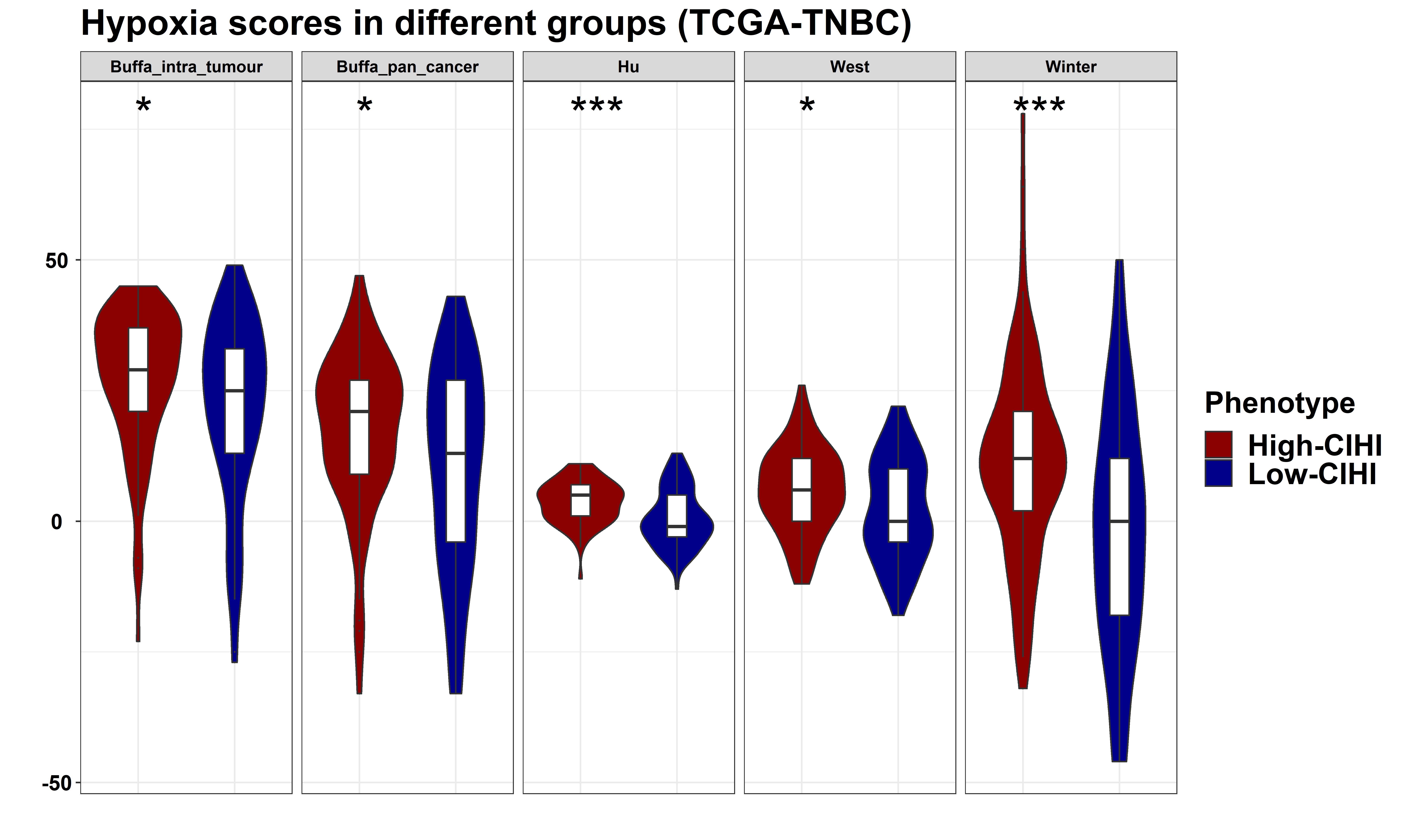

Supplement: Supplementary file 9 — Fig S9. Correlation between the CIHI and previously reported hypoxia scores in the TCGA‐TNBC cohort. [file MOL2-14-2814-s009.tif]
