# Supplementary material for: Identification and validation of a combined hypoxia and immune index for triple‐negative breast cancer
Source: Mol Oncol. 2020 Jul 1;14(11):2814–33. doi: 10.1002/1878-0261.12747 (PMC7607163; doi:10.1002/1878-0261.12747)
Supplement: Supplementary file 10 — Table S1. Primers used in this study. [file MOL2-14-2814-s010.docx]

Table S1. Primers used in this study.

| Gene | F | R |
| --- | --- | --- |
| SERPINE1 | AGTGGACTTTTCAGAGGTGGA | GCCGTTGAAGTAGAGGGCATT |
| TAPBPL | TGCCCGGCCTCACTATACA | GCTCAGTCGTACTTTAGGGGAAG |
| TANK | AGCAGAGAATACGTGAACAACAG | CAGAAGCAATGTCTACCTTTGGT |
| IL12B | ACCCTGACCATCCAAGTCAAA | TTGGCCTCGCATCTTAGAAAG |
| CXCL11 | GAGTGTGAAGGGCATGGCTA | ATAAGCCTTGCTTGCTTCGAT |
| HLA-A | ACCAGGAGACACGGAATGTG | CCATCCAGGTAGGCTCTCAAC |
| PFKL | GCTGGGCGGCACTATCATT | TCAGGTGCGAGTAGGTCCG |
| IL18RAP | ATGCTCTGTTTGGGCTGGATA | GTGAGAGTCGATTTCTGTGGC |
| SLC25A1 | ACGGGGTTAGGGAGATTGTG | GCCTGCAATAGCTCCGAAGA |
| TCF7L2 | AGAAACGAATCAAAACAGCTCCT | CGGGATTTGTCTCGGAAACTT |
| β-actin | AATCGTGCGTGACATTAAGGAG | ACTGTGTTGGCGTACAGGTCTT |
