# Supplementary material for: Identification and validation of a combined hypoxia and immune index for triple‐negative breast cancer
Source: Mol Oncol. 2020 Jul 1;14(11):2814–33. doi: 10.1002/1878-0261.12747 (PMC7607163; doi:10.1002/1878-0261.12747)
Supplement: Supplementary file 11 — Table S2. Reagents used in this study. [file MOL2-14-2814-s011.docx]

Table S2. Reagents used in this study.

| Reagents | Source | Application |
| --- | --- | --- |
| Anti-PD-L1 antibody | Proteintech | 1:100 for IF |
| Anti-CA-IX antibody | Proteintech | 1:100 for IF |
| DAPI | Beyotime | 5μg/ml |
| Antigen Retrieval Solution | CWBIO | 1:100 |
| Alexa Fluor 594 Goat Anti-Mouse IgG (H+L) | Invitrogen | 1:3000 |
| Alexa Fluor 488 Goat Anti-Rabbit IgG (H+L) | Invitrogen | 1:3000 |
| DAB | Dako | Ready for use |
